# Treatment patterns and long-term outcomes in anti-VEGF-treated macular oedema secondary to retinal vein occlusion: a retrospective observational study

**DOI:** 10.1038/s41433-025-04089-2

**Published:** 2025-11-14

**Authors:** Christiana Dinah, Melanie Dodds, Andrew Lotery, Serena Salvatore, Emily Fletcher, Alice V. R. Lake, Antony Parker, Liliana Paris Pereira, Anne-Cecile Retiere, Insaf Saffar, Pablo Arrisi, United Kingdom Retinal Vein Occlusion Electronic Medical Records Users Group (UK RVO EMR Users Group), Indra Dias, Richard Antcliff, Faruque Ghanchi, James Talks, Chris Brand, Aires Lobo, Louise Downey, Colin Jones, Sheena George, Ashish Patwardhan, Yinka Osoba, Balasubramanian Ramasamy, Gloria C. Chi

**Affiliations:** 1 London North West University Healthcare NHS Trust, London, UK; 2 Medisoft, Leeds, UK; 3 University of Southampton, Faculty of Medicine, Southampton, UK; 4 Gloucestershire Hospitals NHS Foundation Trust, Gloucester, UK; 5 University Hospitals of Bristol and Weston, Bristol, UK; 6 Roche Products Ltd, Welwyn Garden City, UK; 7 Genentech, Inc., San Francisco, CA USA; 8 Calderdale and Huddersfield NHS Foundation Trust, Huddersfield, UK; 9 Royal United Hospital Bath NHS Trust, Bath, UK; 10 Bradford Teaching Hospitals NHS Foundation Trust, Bradford, UK; 11 Newcastle Upon Tyne Hospitals NHS Foundation Trust, Newcastle Upon Tyne, UK; 12 Sheffield Teaching Hospitals NHS Foundation Trust, Sheffield, UK; 13 Bedfordshire Hospitals NHS Foundation Trust, Luton, UK; 14 Hull University Teaching Hospitals NHS Trust, Hull, UK; 15 Norfolk and Norwich University Hospitals NHS Foundation Trust, Norwich, UK; 16 The Hillingdon Hospital NHS Foundation Trust, Uxbridge, UK; 17 Royal Cornwall Hospitals NHS Trust, Truro, UK; 18 Torbay and South Devon NHS Foundation Trust, Torquay, UK; 19 Wirral University Teaching Hospital NHS Foundation Trust, Wirral, UK

**Keywords:** Health care, Outcomes research

## Abstract

**Background/objectives:**

This study reported real-world visual acuity (VA), treatment patterns, and ocular safety outcomes from patients with macular oedema secondary to branch, central, or hemi-retinal vein occlusion (BRVO, CRVO, HRVO) treated with anti-vascular endothelial growth factor (VEGF).

**Methods:**

Data were collected from Medisoft electronic medical records across 16 NHS ophthalmology sites in England over 60 months.

**Results:**

3511/3465 eyes/patients with BRVO and 3568/3514 eyes/patients with CRVO or HRVO were included; <2% of patients had bilateral RVO and had both eyes included. VA change from index anti-VEGF treatment was lower with longer follow-up. In BRVO eyes, mean (95% CI) change in VA from index was +11.2 (10.6, 11.7) approximate Early Treatment Diabetic Retinopathy Study (ETDRS) letters at Month (M)6 and +8.3 (6.7, 10.0) at M60. Mean (95% CI) change in VA for CRVO/HRVO eyes was +11.5 (10.7, 12.3) approximate ETDRS letters at M6 and +7.0 (4.8, 9.2) at M60. Eyes receiving more injections displayed greater average VA gains. Median (Q1–Q3) annual number of anti-VEGF injections was lower in BRVO and CRVO/HRVO eyes with longer follow-up: M12, 7.0 (5.0–8.0) and 6.0 (4.0–8.0); M60, 2.0 (0.0–5.0) and 1.0 (0.0–5.0). Ocular safety outcome incidence through 60 months was low (endophthalmitis, retinal tears, and retinal detachment <1%).

**Conclusions:**

VA improvements were observed soon after anti-VEGF treatment, but were lower with longer follow-up through 60 months, alongside lower annual injection numbers, suggesting that current single-target intravitreal anti-VEGFs may not be sufficiently durable for long-term RVO management in routine practice.

## Plain language summary

Retinal vein occlusion (RVO) happens when a vein in the retina is blocked. It affects vision, which can worsen unless treated. One type of treatment, called anti-VEGF medication, is given by regular injection into the eye over a long period of time. This study investigated vision changes over 5 years in patients with RVO, treated with anti-VEGF medications. The study also looked at side effects after treatment and how patients were treated, for example the number of injections they received each year and whether they received other medications. The results showed that anti-VEGF medications are effective for RVO, but need to be delivered frequently to maintain vision gained in the long-term, and that treatments that improve vision in the long-term are still needed for patients with RVO.

## Introduction

Retinal vein occlusion (RVO) can lead to vision impairment and is the second most common retinovascular disease after diabetic retinopathy [1]. Prevalence of RVO is strongly correlated with age, hypertension, smoking, hyperlipidaemia, arteriolosclerosis, and hypercholesterolaemia [2, 3]. Although the pathogenesis of RVO remains poorly characterised, a combination of vascular and inflammatory mediators contribute to its complexity [4, 5]. Macular oedema secondary to RVO occurs when vascular occlusion restricts blood flow exiting the retina, increasing capillary pressure, causing fluid leakage into the macula [6]. Along with retinal ischaemic changes this causes vision impairment, including severe vision loss in extreme cases [3].

Pharmacological treatments aiming to reduce retinal leakage to prevent or reverse vision impairment include therapies targeting the vascular endothelial growth factor (anti-VEGF treatments), and steroids [7]. These therapies are administered intravitreally (IVT) in routine clinical care. Nonpharmacological therapies are also used, such as focal or macular grid laser photocoagulation [8, 9]. In routine clinical care, anti-VEGF treatments are administered according to pro-re-nata (PRN) or treat-and-extend (T&E) regimens [10]. To ensure long-term disease control whilst accommodating for RVO treatment burden, continued monitoring of treatment outcomes should ideally be personalised [10, 11].

Real-world studies suggest that visual acuity (VA) outcomes in patients with RVO are often poorer than in clinical trial populations, partly due to the significant treatment and monitoring burden associated with administering frequent IVT injections and the demand on healthcare providers, patients, and caregivers in the treatment of retinal vascular disorders [11–13]. This highlights the need for a deeper understanding of real-world treatment outcomes and patterns to identify areas for improvement in the long-term treatment of macular oedema secondary to RVO.

### Objectives

This study aimed to describe real-world VA outcomes in eyes with macular oedema secondary to RVO for up to 60 months after initiation of anti-VEGF therapy, in addition to real-world treatment patterns, and the incidence of pre-defined ocular safety outcomes within 60 months of index anti-VEGF treatment.

## Methods

### Study design

This study was a retrospective, observational, descriptive study of anonymised Medisoft electronic medical record (EMR) data from 16 NHS ophthalmology sites in England.

Eyes with a diagnosis of macular oedema secondary to branch (BRVO), central RVO (CRVO), or hemi-RVO (HRVO) were identified using Medisoft’s proprietary diagnosis library. All data for these eyes were anonymised and extracted from sites between 23 and 29 August 2023, after local Medical Retina lead and Caldicott Guardian approval.

### Study population

Inclusion criteria were eyes from patients aged ≥18 years, with an initial diagnosis of macular oedema secondary to one of BRVO, CRVO, or HRVO between January 2013 and August 2023, and treated with at least one anti-VEGF injection on or after diagnosis. Initial diagnosis date was the first diagnosis of macular oedema secondary to RVO during the study period per eye. A VA measurement was required ≤30 days prior to or on the index treatment date, defined as the first anti-VEGF treatment an eye received following initial diagnosis. Eyes needed a minimum of 3 months’ follow-up post index treatment date. Both eyes of a patient were included if they satisfied the inclusion criteria.

Eyes were excluded if they had a diagnosis of both BRVO *and* CRVO, or BRVO *and* HRVO; if they had received prior treatment with anti-VEGF, IVT steroids, vitrectomy, or laser; or if they had separate diagnoses of any of the following conditions on or before initial diagnosis: moderate/worse proliferative or non-proliferative diabetic retinopathy (DR), diabetic macular oedema, neovascular age-related macular degeneration (nAMD), geographic atrophy, myopic choroidal neovascularisation, cystoid macular oedema, or retinal neovascularisation. To capture real-world RVO treatment patterns, eyes that initiated supplemental treatment with IVT steroids, vitrectomy or laser after index were not excluded or censored at the point of initiating the supplemental treatment.

Data for CRVO and HRVO are reported together for all analyses (CRVO/HRVO; n = 3514); BRVO results (n = 3465) are reported separately. No formal sample size calculation was performed as the sample was collected from all available and eligible medical records.

### Study measures

#### Vision outcomes

VA change and descriptive summaries of the VA at each follow-up timepoint from index treatment were assessed through 60 months; VA change was stratified by baseline VA (VA at index treatment), number of anti-VEGF injections received through 60 months, and by completion of the loading phase (loading dose of ≥3 anti-VEGF injections within the first 120 days of index treatment, including the index treatment injection).

Where recorded in Snellen or logMAR, VA was converted to an approximate Early Treatment Diabetic Retinopathy Study (ETDRS) letter score; calculations are provided in the Supplementary Methods [14], and the baseline distribution of the number of eyes originally assessed using Snellen, LogMAR and ETDRS letter score is presented in Supplementary Table 1. Baseline VA was stratified using approximate ETDRS letter thresholds whilst number of anti-VEGF injections was stratified by tertiles (Supplementary Methods).

When capturing VA at different timepoints, a buffer was allowed on either side of the date to accommodate for any deviation in real-world treatment appointments and increase the chances of each eye having a recorded value. For an eye that had two readings inside the buffer window, the reading closest to the date of interest was taken. If the two readings were equidistant, the earlier of the two was used. The buffers were:

- Index Date: −30 days
- Follow-up reading at Month 3, 6, 12, or 18: ±30 days
- Follow-up reading at Month 24, 36, 48 or 60: ±60 days

#### Treatment patterns

Treatment by IVT injection (anti-VEGF or steroid) was defined as the Office of Population Censuses and Surveys Classification of Interventions and Procedures (OPCS-4) code C79.4. Treatment by macular or grid laser was also identified using OPCS-4 codes. Masked use of anti-VEGF agents in any clinical trials were not included.

As included eyes required ≥3 months’ follow-up, the sample may be skewed towards patients with higher socioeconomic status compared with the overall population of patients with macular oedema secondary to RVO. Patients without care responsibilities and with access to private transport are more likely to return to clinic more regularly, as are those whose caregivers do not have long-term work commitments or can more easily take time off for patients’ treatment. To examine the relationship between socioeconomic status and treatment patterns, VA at index treatment and the number of anti-VEGF injections received were summarised by Index of Multiple Deprivation (IMD) deciles based on residential postcodes, according to the English Indices of Deprivation (https://www.gov.uk/government/statistics/english-indices-of-deprivation-2015). A lower IMD decile represents greater neighbourhood-level deprivation. In this study, IMD deciles were analysed as 5 categories of 2 deciles each.

#### Safety outcomes

Cumulative incidences of pre-defined ocular safety outcomes of interest were tracked and analysed over 60 months of index treatment. Safety outcome counts reported are cumulative over Months 3–60 and are not mutually exclusive over timepoints (i.e. the same safety outcome may be recorded multiple times in a patient at different timepoints). Pre-index occurrences for each safety outcome were also identified from patients’ history and are reported for context; pre-index safety events were not included in the cumulative count of post-index safety events.

Pre-defined ocular safety outcomes were: ocular hypertension, glaucoma, endophthalmitis, other intraocular inflammation (IOI; including uveitis, vitritis, iridocyclitis, chorioretinitis, anterior chamber flare/inflammation, retinal vasculitis, vitreous cells, and iritis), retinal detachment, retinal tear, vitreous haemorrhage, traumatic cataract, cataract surgery, retinal artery occlusion (RAO), possible progression of retinal ischaemia (rubeosis, retinal neovascularisation, vision loss >15 approximate ETDRS letters).

Severe outcomes of interest in this analysis were the occurrence of the following within 30 days of the safety outcome: sudden vision loss (≥30 approximate ETDRS letters) following treatment, and the need for surgical intervention, which included trabeculoplasty, iridotomy, trabeculectomy, vitrectomy, cryopexy, retinopexy, photocoagulation, and cyclophotocoagulation, dependent on the safety outcome.

### Data analysis

Data modelling was conducted using the application Structured Query Language (SQL) Server Management Studio (SSMS) and data were analysed using the statistical software R with the integrated development environment RStudio (SQL Server version: SQL Server 2019 Enterprise; SSMS version: SQL Server Management Studio version: 19; R version: 4.3.0; RStudio version: 2023.06.1+524) [15]. For patients with bilateral disease (both eyes qualifying against the inclusion criteria), data for both eyes were included. For specific timepoints, data could be missing if there were no recorded data for the eye during the time window of interest (e.g. 12 months ±30 days). Missing data were not imputed. Mean values with standard deviation (SD) or 95% confidence intervals (95% CI) are presented throughout.

## Results

### Baseline demographics and eye characteristics

After applying inclusion criteria, 3511 eyes from 3465 patients with BRVO and 3568 eyes from 3514 patients with CRVO/HRVO were included (Table 1, Supplementary Fig. 1). A summary of the anti-VEGF agents used and their prevalence across BRVO and CRVO/HRVO is provided in Supplementary Table 2; patients were characterised as having received aflibercept only (Eylea^®^ 2 mg), ranibizumab only (Lucentis^®^), or mixed or other anti-VEGFs.

**Table 1:** Patient demographics and eye characteristics.

Patient demographics		BRVO		CRVO/HRVO
Number of *patients* with macular oedema secondary to BRVO/CRVO/HRVO between 2013–2023		3465		3514
Age at index treatment (years)^a^				
Mean (SD)		71.5 (12.2)		73.6 (12.4)
Sex, n (%)				
Female		1852 (53.5)		1671 (47.6)
Male		1613 (46.6)		1843 (52.5)
Race/ethnicity, n (%)				
White British		2761 (79.7)		2923 (83.2)
White Irish		19 (0.6)		15 (0.4)
Asian or Asian British		85 (2.5)		74 (2.1)
Black or Black British		20 (0.6)		27 (0.8)
Chinese		4 (0.12)		1 (0.03)
Any other White background		66 (1.9)		41 (1.2)
Any other mixed background		7 (0.2)		8 (0.2)
Any other ethnic group		20 (0.6)		18 (0.5)
Not stated		483 (13.9)		407 (11.6)
IMD decile, n (%)				
1 (most deprived)		264 (7.6)		307 (8.7)
2		234 (6.8)		248 (7.1)
3		252 (7.3)		292 (8.3)
4		330 (9.5)		337 (9.6)
5		340 (9.8)		334 (9.5)
6		390 (11.3)		382 (10.9)
7		400 (11.5)		386 (11.0)
8		396 (11.4)		383 (10.9)
9		362 (10.5)		360 (10.2)
10 (least deprived)		479 (13.8)		461 (13.1)
Not stated		18 (0.5)		24 (0.7)

Eye characteristics		BRVO		CRVO/HRVO
Number of *eyes* with macular oedema secondary to BRVO/CRVO/HRVO between 2013–2023		3511		3568
Laterality, n (%)				
Left		1750 (49.8)		1716 (48.1)
Right		1761 (50.2)		1852 (51.9)
Lens status, n (%)				
Aphakic		10 (0.3)		8 (0.2)
Phakic		2619 (74.6)		2752 (77.1)
Pseudophakic		706 (20.1)		745 (20.9)
Not known		176 (5.0)		63 (1.8)
Year of diagnosis, n (%)				
2013		64 (1.8)		84 (2.4)
2014		198 (5.6)		188 (5.3)
2015		290 (8.3)		286 (8.0)
2016		330 (9.4)		369 (10.3)
2017		328 (9.3)		371 (10.4)
2018		389 (11.1)		409 (11.5)
2019		396 (11.3)		384 (10.8)
2020		377 (10.8)		404 (11.3)
2021		506 (14.4)		444 (12.4)
2022		461 (13.1)		476 (13.3)
2023		172 (4.9)		153 (4.3)
Duration since diagnosis at index treatment (days)^a^				
Median (Q1–Q3)		0 (0–14)		0 (0–14)
Duration of follow-up after index treatment, n (%)^a^				
≥3 months (≥90 days)		3511 (100.0)		3568 (100.0)
≥6 months (≥180 days)		3275 (93.3)		3326 (93.2)
≥12 months (≥360 days)		2856 (81.3)		2879 (80.7)
≥18 months (≥540 days)		2425 (69.1)		2425 (68.0)
≥24 months (≥720 days)		2007 (57.2)		2055 (57.6)
≥36 months (≥1080 days)		1369 (39.0)		1420 (39.8)
≥48 months (≥1440 days)		1012 (28.8)		993 (27.8)
≥60 months (≥1800 days)		673 (19.2)		679 (19.0)
VA at index treatment (approximate ETDRS letter score)^a^ i.e. analysis groups				
≤35 letters		564 (16.1)		1494 (41.9)
≥70 letters		908 (25.9)		412 (11.6)
≥85 letters		61 (1.7)		22 (0.6)
VA at index treatment (approximate ETDRS letter score)^a^				
Median (Q1–Q3)		60 (46–70)		46 (20–61)

Demographic data are reported at the individual patient level; eye characteristics are reported at the individual eye level.

*BRVO* branch retinal vein occlusion, *CRVO* central retinal vein occlusion, *ETDRS* Early Treatment Diabetic Retinopathy Study, *HRVO* hemiretinal vein occlusion, *IMD* Index of Multiple Deprivation, *Q1* lower quartile, *Q3* upper quartile, *SD* standard deviation, *VA* visual acuity.

^a^Index treatment defined as the first anti-VEGF treatment an eye received following initial diagnosis.

Median (Q1–Q3) duration from initial diagnosis to index treatment date was 0 (0–14.0) days for all eyes (Table 1). Over 55% of eyes had at least 24 months’ follow-up after index treatment (BRVO: 57.2%; CRVO/HRVO: 57.6%) whilst <20% were followed up for 60 months (BRVO: 19.2%; CRVO/HRVO: 19.0%). Mean (SD) index VA was 55.3 (17.9) approximate ETDRS letters for BRVO eyes and 39.5 (24.3) approximate ETDRS letters for CRVO/HRVO eyes.

Mean index VA was generally similar for eyes across IMD deciles but was on average ~4–5 letters lower for IMD deciles 1–2 (most deprived) compared to deciles 9–10 (least deprived) (Supplementary Fig. 2). Over the total duration of follow-up, for BRVO, 38.6% of eyes and 37.6% of eyes were lost to follow-up for IMD deciles 1–2 and 9–10, respectively. For CRVO/HRVO eyes, 49.7% of eyes were lost to follow-up for IMD deciles 1–2 compared with 39.3% for IMD deciles 9–10 (Supplementary Fig. 3).

Baseline demographics and characteristics for eyes with and without VA data at Month 60 are provided in Supplementary Tables 3a, b. Mean (SD) index VA was slightly higher in eyes with versus without VA data at Month 60 for both BRVO (58.0 [15.3] vs 54.8 [18.2]) and CRVO/HRVO (43.8 [23.5] vs 38.8 [24.3]) eyes. Mean (SD) age was slightly lower in eyes with versus without VA data at Month 60 for both BRVO (69.4 [10.1] vs 71.8 [12.5] years) and CRVO/HRVO (71.5 [10.4] vs 74.0 [12.6] years) eyes.

### Visual outcomes

#### VA change from index date

**Fig. 1:**
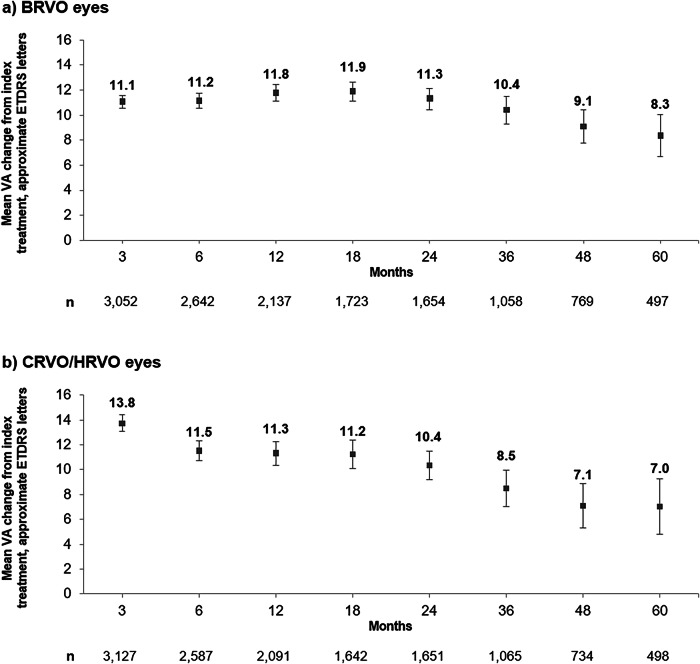
VA change from index anti-VEGF treatment over 60 months. **a** BRVO eyes. **b** CRVO/HRVO eyes. Error bars represent 95% confidence intervals; n numbers represent the number of eyes with a VA measurement at index and each individual time point of interest. BRVO branch retinal vein occlusion, CRVO central retinal vein occlusion, ETDRS Early Treatment Diabetic Retinopathy Study, HRVO hemiretinal vein occlusion, VA visual acuity.

VA gain from index treatment was comparatively lower with longer duration of follow-up, noting that each follow-up cohort included a varying number of eyes due to loss to follow-up, or the recency of eyes entering the study. For BRVO eyes, mean (95% CI) VA gain from index treatment in approximate ETDRS letters was 11.2 (10.6, 11.7) at Month 6, 11.8 (11.1, 12.5) at Month 12, and 8.3 (6.7, 10.0) at Month 60 (Fig. 1a). For CRVO/HRVO eyes, VA gain in approximate ETDRS letters was 11.5 (10.7, 12.3) at Month 6, 11.3 (10.4, 12.3) at Month 12, and 7.0 (4.8, 9.2) at Month 60 (Fig. 1b).

Overall, ~80% of eyes were lost to follow-up by Month 60. Among BRVO eyes with VA data at Months 6 and 60 (n = 449 [66.7% of eligible eyes at Month 60]), mean (SD) VA was 58.4 (15.2) at index and 69.6 (12.1) at Month 6, representing a mean (95% CI) VA gain of 11.2 (9.9, 12.4) letters, comparable to the gains seen in the overall cohort. In CRVO/HRVO eyes with VA data at Months 6 and 60 (n = 412 [60.7%]), mean (SD) was 44.0 (23.2) at index and 55.8 (21.7) at Month 6, demonstrating a mean (95% CI) VA gain of 11.8 (10.0, 13.6) letters, also comparable to the gains seen in the overall cohort.

**Fig. 2:**
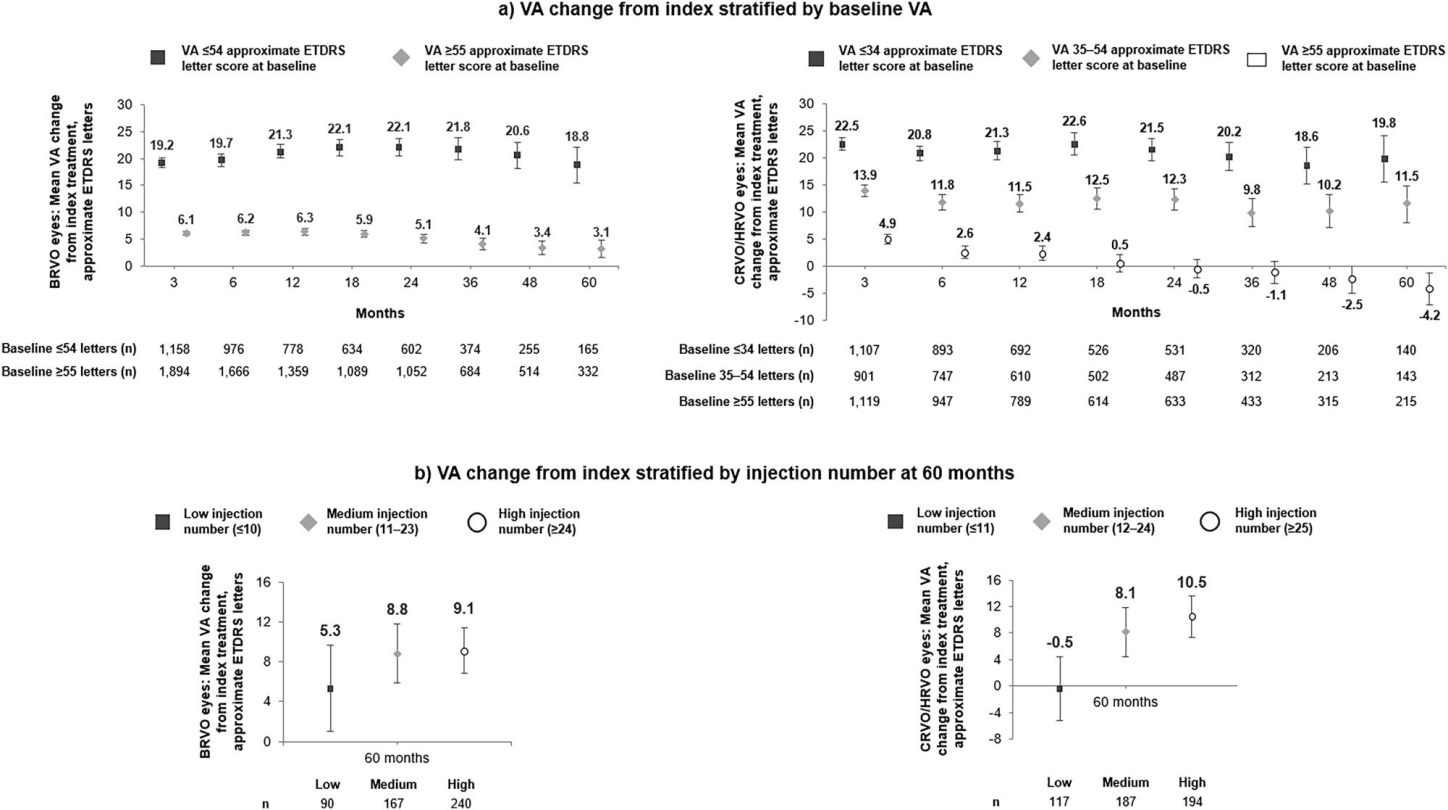
VA change from index injection in BRVO and CRVO/HRVO eyes. **a** VA change from index stratified by baseline VA over 60 months. **b** VA change from index stratified by injection number at 60 months. Error bars represent 95% confidence intervals; n numbers represent the number of eyes with a VA measurement at index and each individual time point. BRVO branch retinal vein occlusion, CRVO central retinal vein occlusion, ETDRS Early Treatment Diabetic Retinopathy Study, HRVO hemiretinal vein occlusion, VA visual acuity.

When stratified by VA at baseline, mean VA gain was greater at all timepoints in eyes with lower baseline VA, and comparatively lower in eyes with a better VA at baseline (Fig. 2a). Greater VA improvements at 60 months were also observed in eyes that received a high number of injections over 60 months (≥24 injections for BRVO; ≥25 for CRVO) than eyes that received a low number of injections (≤10 for BRVO and ≤11 for CRVO) (Fig. 2b).

On average, greater VA gains from index treatment to Month 3 through Month 60 were observed in eyes that completed the loading phase (Supplementary Fig. 4).

#### Maintenance of VA

Among BRVO eyes with VA ≥70 approximate ETDRS letters at index (n = 908 [25.9%]), 590/683 (86.4%) maintained VA ≥70 approximate ETDRS letters at 6 months post-index treatment, 488/565 (86.4%) at 12 months, and 99/138 (71.7%) at 60 months. In CRVO/HRVO eyes with VA ≥70 approximate ETDRS letters at index (n = 412 [11.6%]), 229/300 (76.3%) maintained VA ≥70 approximate ETDRS letters at 6 months, 172/238 (72.3%) at 12 months, and 38/71 (53.5%) at 60 months (Supplementary Fig. 5).

#### VA gains and avoidance of loss

At Month 12, VA gains of ≥15 ETDRS letters were observed in 865/2137 (40.5% [95% CI: 38.4%, 42.6%]) of BRVO and 965/2091 (46.2% [44.0%, 48.3%]) CRVO/HRVO eyes, respectively, with avoidance of loss of ≥15 letters observed in 2060/2137 (96.4% [95.5%, 97.1%]) and 1876/2091 (89.7% [88.3%, 91.0%]) eyes, respectively. At Month 60, VA gains of ≥15 letters were still observed in 193/497 (38.8% [34.7%, 43.2%]) BRVO eyes and 197/498 (39.6% [35.4%, 43.9%]) CRVO/HRVO eyes, with avoidance of loss of ≥15 letters observed in 454/497 (91.3% [88.6%, 93.5%]) BRVO eyes and 406/498 (81.5% [77.9%, 84.7%]) CRVO/HRVO eyes (Supplementary Figs. 6 and 7).

### Treatment patterns

Of the 3511 BRVO and 3568 CRVO/HRVO eyes entering the study, 1374 (39.1%) and 1518 (42.5%), respectively, were lost to follow-up or had non-persistence of treatment over the 60-month period.

**Fig. 3:**
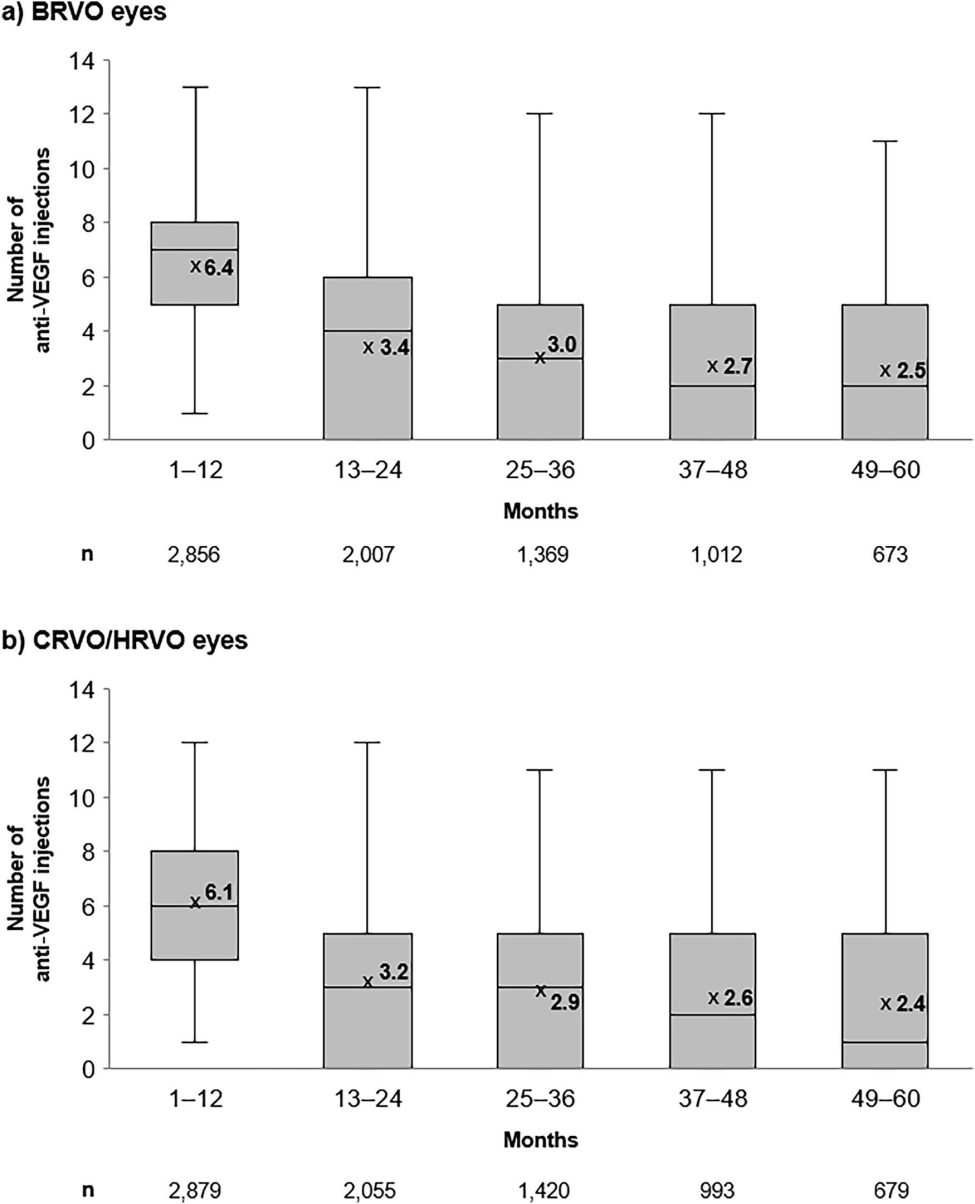
Number of anti-VEGF injections received through 60 months. **a** BRVO eyes. **b** CRVO/HRVO eyes. x represents the mean value; n numbers represent the number of eligible eyes at each time point. BRVO branch retinal vein occlusion, CRVO central retinal vein occlusion, HRVO hemiretinal vein occlusion, VEGF vascular endothelial growth factor.

BRVO and CRVO/HRVO eyes received a mean (SD) of 4.3 (1.2) and 4.1 (1.1) anti-VEGF injections from Months 0–6 and 2.1 (1.5) and 2.0 (1.5) injections from Months 7–12, respectively. Median (Q1–Q3) numbers of anti-VEGF injections were 4.0 (3.0–5.0) in both BRVO and CRVO/HRVO cohorts from Months 0–6 and 2.0 (1.0–3.0) in both cohorts from Months 7–12. Eyes received, on average, less than 3 injections from Months 49–60. Both mean and median number of anti-VEGF injections received per year was lower with longer follow-up for all eyes (Fig. 3), whilst the percentage of eyes that received IVT steroids or macular laser was higher with longer follow-up (Supplementary Fig. 8). The number of anti-VEGF injections received was similar across timepoints in eyes of patients from different IMD deciles (Supplementary Fig. 9).

### Safety

Over the 60-month follow-up period, at least one of the pre-defined safety outcomes was reported in 11.0% of BRVO and 17.2% of CRVO/HRVO eyes; 1.1% of BRVO and 2.7% of CRVO/HRVO eyes reported more than one pre-defined safety outcome.

Cumulative post-index incidences of pre-defined safety outcomes of interest are reported in Table 2; pre-index occurrences are also reported for context. At 6 months after index treatment, the cumulative incidences of IOI were low, at 0.1% in BRVO eyes and 0.2% in CRVO/HRVO eyes. Cumulatively through Month 60, this was 0.2% in BRVO and 1.3% in CRVO/HRVO eyes. Across all timepoints, the incidence of ocular hypertension remained below 5% and the incidence of glaucoma remained below 12% in all eyes. Similarly, across all timepoints, incidences of endophthalmitis (<0.6%), retinal tear (<0.8%), retinal artery occlusion (<0.8%) and retinal detachment (<0.8%) were low for both BRVO and CRVO/HRVO eyes. Rubeosis was observed in <0.2% of eyes with BRVO and <1.3% of eyes with CRVO/HRVO at all follow-up timepoints of interest. No traumatic cataract was reported in BRVO or CRVO/HRVO eyes at all follow-up timepoints.

**Table 2:** Safety outcomes for eyes with BRVO and CRVO/HRVO.

	BRVO Eyes									CRVO/HRVO Eyes								
Follow-up time point	Pre-index	3 months	6 months	12 months	18 months	24 months	36 months	48 months	60 months	Pre-index	3 months	6 months	12 months	18 months	24 months	36 months	48 months	60 months
Eligible eyes, n	3511	3511	3275	2856	2425	2007	1369	1012	673	3568	3568	3326	2879	2425	2055	1420	993	679
Ocular hypertension	51 (1.45)	6 (0.17)	8 (0.24)	16 (0.56)	24 (0.99)	23 (1.15)	20 (1.46)	23 (2.27)	19 (2.82)	147 (4.12)	15 (0.42)	22 (0.66)	36 (1.25)	45 (1.86)	46 (2.24)	48 (3.38)	43 (4.33)	30 (4.42)
Glaucoma	233 (6.64)	24 (0.68)	30 (0.92)	45 (1.58)	54 (2.23)	57 (2.84)	58 (4.24)	57 (5.63)	47 (6.98)	359 (10.06)	52 (1.46)	64 (1.92)	91 (3.16)	104 (4.29)	112 (5.45)	110 (7.75)	91 (9.16)	77 (11.34)
Vitreous haemorrhage	14 (0.40)	0	0	0	3 (0.12)	4 (0.20)	5 (0.37)	5 (0.49)	4 (0.59)	17 (0.48)	17 (0.48)	21 (0.63)	21 (0.73)	24 (0.99)	22 (1.07)	23 (1.62)	19 (1.91)	16 (2.36)
Cataract, n (%)																		
Traumatic cataract	1 (0.03)	0	0	0	0	0	0	0	0	0	0	0	0	0	0	0	0	0
Cataract surgery	279 (7.95)	8 (0.23)	21 (0.64)	50 (1.75)	70 (2.89)	80 (3.99)	72 (5.26)	73 (7.21)	67 (9.96)	301 (8.44)	8 (0.22)	14 (0.42)	41 (1.42)	66 (2.72)	85 (4.14)	101 (7.11)	85 (8.56)	68 (10.01)
IOI, n (%)																		
IOI (overall)^a^	14 (0.40)	3 (0.09)	4 (0.12)	4 (0.14)	4 (0.16)	4 (0.20)	2 (0.15)	1 (0.10)	1 (0.15)	9 (0.25)	3 (0.08)	5 (0.15)	6 (0.21)	8 (0.33)	7 (0.34)	9 (0.63)	9 (0.91)	9 (1.33)
Endophthalmitis	0	1 (0.03)	1 (0.03)	2 (0.07)	3 (0.12)	4 (0.20)	4 (0.29)	3 (0.30)	3 (0.45)	0	3 (0.08)	4 (0.12)	4 (0.14)	5 (0.21)	6 (0.29)	4 (0.28)	4 (0.40)	4 (0.59)
Retinal artery occlusion, n (%)																		
RAO (overall)^b^	8 (0.23)	1 (0.03)	4 (0.12)	7 (0.25)	9 (0.37)	9 (0.45)	7 (0.51)	5 (0.49)	3 (0.45)	7 (0.20)	5 (0.14)	7 (0.21)	6 (0.21)	7 (0.29)	8 (0.39)	5 (0.35)	6 (0.60)	5 (0.74)
Retinal trauma, n (%)																		
Retinal tear	1 (0.03)	0	1 (0.03)	1 (0.04)	3 (0.12)	4 (0.20)	4 (0.29)	5 (0.49)	5 (0.74)	4 (0.11)	1 (0.03)	1 (0.03)	2 (0.07)	3 (0.12)	3 (0.15)	3 (0.21)	2 (0.20)	2 (0.29)
Retinal detachment	8 (0.23)	1 (0.03)	2 (0.06)	3 (0.11)	5 (0.21)	7 (0.35)	6 (0.44)	5 (0.49)	5 (0.74)	5 (0.14)	0	1 (0.03)	2 (0.07)	2 (0.08)	2 (0.10)	4 (0.28)	2 (0.20)	3 (0.44)
Possible progression of retinal ischaemia, n (%)																		
Rubeosis (iris neovascularisation)	0	0	0	0	0	0	1 (0.07)	1 (0.10)	1 (0.15)	9 (0.25)	23 (0.64)	24 (0.72)	30 (1.04)	30 (1.24)	25 (1.22)	20 (1.41)	10 (1.01)	3 (0.44)
Retinal neovascularisation	2 (0.06)	1 (0.03)	2 (0.06)	4 (0.14)	6 (0.25)	6 (0.30)	7 (0.51)	5 (0.49)	3 (0.45)	2 (0.06)	2 (0.06)	3 (0.09)	3 (0.10)	4 (0.16)	3 (0.15)	3 (0.21)	4 (0.40)	3 (0.44)
Vision loss >15 ETDRS letters^c^	0	33 (0.94)	41 (1.25)	43 (1.51)	48 (1.98)	41 (2.04)	41 (2.99)	29 (2.87)	23 (3.42)	0	78 (2.19)	95 (2.86)	93 (3.23)	88 (3.63)	80 (3.89)	67 (4.72)	50 (5.04)	29 (4.27)

Data are reported as the percentage of eyes with eligible data at each time point with a recorded safety outcome of interest. Safety outcome counts from Month 3 to Month 60 are cumulative over follow-up time and are not mutually exclusive between timepoints; i.e. the same safety outcome may be recorded multiple times in a patient at different timepoints. Losses to follow-up accounts for the fluctuations in safety outcome counts observed through 60 months. Pre-index safety outcome events were not included in the cumulative post-index safety outcome count.

*BRVO* branch retinal vein occlusion, *CRVO* central retinal vein occlusion, *ETDRS* Early Treatment Diabetic Retinopathy Study, *HRVO* hemiretinal vein occlusion, *IOI* intraocular inflammation, *RAO* retinal artery occlusion.

^a^Includes uveitis, vitritis, iridocyclitis, chorioretinitis, anterior chamber flare/inflammation, retinal vasculitis, vitreous cells, iritis.

^b^RAO (overall) includes branch and central RAO.

^c^Not all incidences of vision loss >15 letters were in relation with progression of retinal ischaemia.

#### Severe outcomes

The incidence of severe outcomes following studied safety outcomes is summarised in Supplementary Tables 4a, b.

Among anti-VEGF-treated eyes, severe outcomes were most consistently associated with retinal detachment, retinal tears, endophthalmitis, and vitreous haemorrhage. By 60 months, 100% of cases of retinal detachment were associated with intervention (surgical repair). At 60 months, severe outcomes (transient sudden vision loss or need for surgical intervention) had occurred after at least 50.0% of retinal tears, at least 37.5% of vitreous haemorrhages, and after all cases of endophthalmitis.

## Discussion

This retrospective, observational, real-world study followed eyes with macular oedema secondary to RVO receiving anti-VEGF treatment across 16 ophthalmology clinics in England for up to 60 months. The results indicate that vision gains achieved with single-target anti-VEGFs are not maintained over the observed duration of follow-up, suggesting that anti-VEGF monotherapy alone may not be sufficiently durable, or provide sustained effectiveness, for long-term management of RVO in routine clinical practice.

Anti-VEGF treatments for macular oedema secondary to RVO resulted in overall positive changes from index VA, with mean VA gains of 11.3–11.8 approximate ETDRS letters (equivalent to 2.3–2.4 lines) at Month 12 and 7.0–8.3 approximate ETDRS letters (equivalent to 1.4–1.7 lines) at Month 60. Although the population providing data at Month 60 was much smaller than the overall cohort, similar mean VA gains were observed in this sub-cohort (11.2–11.8 approximate ETDRS letters) compared with the overall cohort (11.2–11.5 approximate ETDRS letters) at Month 6. At Month 60, nearly 40% of eyes achieved VA gains of ≥15 approximate ETDRS letters and over 80% avoided VA loss of ≥15 letters, defined as a moderate visual loss [16]. Through 60 months, over 70% of BRVO eyes and over 50% of CRVO/HRVO eyes maintained VA ≥70 letters, considered to be driving vision; VA lower than this threshold may significantly impact patients’ quality of life [17, 18].

Most VA gains in BRVO and CRVO/HRVO eyes occurred soon after index anti-VEGF treatment, and eyes continued to benefit from treatment through 60 months, reflecting the chronic nature of RVO [19]. Average VA gains were lower with longer follow-up, although they remained positive at all timepoints. Over time, development of retinal structural damage such as epiretinal membranes could contribute to lower VA gains [20, 21]. In addition, fewer anti-VEGF injections were received with each year of treatment. As greater VA gains were observed in eyes with higher vs lower injection numbers through 60 months, the lower total number of anti-VEGF injections over 60 months could have impacted long-term vs short-term VA gains observed [10]. The results of this study are consistent with those reporting lower VA gains over time corresponding with eyes receiving fewer injections [10].

Greater VA improvements through 60 months were also observed in eyes with lower VA at baseline, consistent with studies in both RVO and nAMD [22–24]. In BRVO eyes, at 12 months, mean (SD) VA change in the lowest and highest baseline VA categories was 21.3 (17.9) and 6.3 (11.5) approximate ETDRS letters, respectively; in CRVO/HRVO, this was 21.3 (22.6) and 2.4 (18.6), respectively, noting that this difference may also have been influenced by the ceiling effect in eyes with higher VA at baseline, with less room for improvement [25].

In this study, VA gains were poorer in eyes that did not complete the anti-VEGF loading phase, underlining the importance of early disease control and the impact of non-adherence on VA outcomes in clinical practice. The relationship between poorer VA outcomes with non-completion of the loading phase, along with the decreasing number of injections over time, suggests the need for more durable treatments in the short- and long-term management of patients with RVO. The increasing use of laser or steroid treatment to Month 60 despite ongoing treatment with anti-VEGF therapies suggests the presence of residual or persistent macular oedema, which may be a result of anti-VEGF failure or undertreatment. Indeed, this would be consistent with the LEAVO study comparing ranibizumab, aflibercept and bevacizumab for macular oedema secondary to CRVO, in which 10.7% and 60.8% of eyes had persistent oedema and recurrent oedema, respectively, after 100 weeks [26]. This highlights the potential need for combination therapies or alternative treatment approaches that target multiple pathways when managing this multifactorial condition in the long term. The inability of anti-VEGF therapies alone to effectively control macular oedema in certain patients may explain the observed decrease in the number of injections over extended follow-up periods. However, VA outcomes specifically associated with concomitant laser or steroid treatment and ideal timing for subsequent shifts to supplemental therapies were not evaluated in this study and warrant further research.

Real-world treatment patterns and outcomes often do not reflect those observed in controlled clinical trials, in which participants tend to have more frequent injections and better VA outcomes [22, 23, 25, 27, 28]. In a real-world environment, treatment for a chronic disease such as RVO can pose a significant burden on patients and caregivers, with frequent clinic visits and long-term, invasive treatments leading to decreased adherence over time [11]. In patients receiving care for other conditions, the overall burden of frequent hospital visits may lead them to discontinue treatment [29]. In addition, the growing incidence of retinal diseases affects the capacity of clinics to deliver consistent treatment over time [30], which may result in undertreatment or potential missed treatments.

Socioeconomic status may also impact patients’ access to treatment. Although eyes of patients across IMD deciles had similar index VA and received a similar number of injections over 60 months, index VA was slightly lower for eyes of patients from the most deprived areas (IMD deciles 1–2) compared with the least deprived areas (IMD deciles 9–10). This may reflect the general trend of greater deprivation being associated with poorer VA at presentation and delayed access to care [31].

The cumulative incidence of pre-defined safety outcomes was low, with each outcome occurring in <1% of eyes at 6 months (when follow-up data were available for most patients), except glaucoma which occurred in 1.9% of CRVO/HRVO eyes, and loss of >15 letters in VA score in 1.3% of BRVO eyes and 2.9% of CRVO/HRVO eyes. Complications in RVO relating to retinal ischaemia can cause glaucoma, which could account for the relatively higher incidence observed in CRVO/HRVO eyes [6]. The higher incidence of pre-defined safety outcomes at later timepoints could be explained by the fact that safety outcome counts were cumulative over timepoints, and that the sample size decreased at each subsequent timepoint. Severe outcomes were more frequent with retinal detachment, retinal tears, endophthalmitis, and vitreous haemorrhage, and were more commonly interventions than sudden vision loss.

A study limitation is that results could have been affected by the UK COVID response policies, during 2020 and 2021, when treatment consistency was impacted considerably. Changes in national treatment guidance also occurred during the 2013–2023 study period [6], but these were not adjusted for during data analysis. In addition, the eye population studied at different timepoints varies, with a smaller population included at later timepoints, limiting comparisons across timepoints. This is also influenced by loss to follow-up and the date at which eyes entered the study thereby restricting the length of follow-up possible. Reasons for loss to follow-up are not available from the EMR, but could be due to comorbidities or death.

Mean index VA was higher in eyes that provided VA data at Month 60 compared to those that did not, by ~3 and ~5 letters in BRVO and CRVO/HRVO eyes, respectively. This suggests that among patients lost to follow-up, there may have been a greater proportion of eyes with severe disease and limited room for VA improvement, with poor response to treatment, or that discontinued due to perceived lack of benefit. Additionally, patients from lower socioeconomic groups, who presented with worse VA at baseline, may have had difficulty attending clinics regularly and thus may have been more likely to be lost to follow-up. Consequently, the observed trends in long-term VA gains, treatment patterns and safety may have been influenced by attrition bias. Furthermore, most eyes without VA data at Month 60 were diagnosed less than 5 years prior to data extraction and therefore could not complete 5 years of follow-up; this coincides with COVID-19 lockdowns and subsequent NHS backlogs, which may have contributed to eyes receiving delayed diagnoses when the disease had progressed, therefore presenting with worse index VA. Given that the majority of patients were no longer followed up by Month 60, the findings should be interpreted with caution, acknowledging the potential underrepresentation of patients with poorer responses or complications.

The incidence of safety outcomes may have been underestimated if outcomes were not recorded in the EMR, or if recorded as free text rather than searchable data, and interventions may not have been documented in the EMR. The lack of imaging data to support the VA changes observed in eyes included in this study also limits the inferences that may be drawn. The analysis of treatment patterns by IMD deciles is limited by the fact that the decile was documented based on patients’ residential postcodes at the time of data extraction, not accounting for any change in residence through the duration of the study. Additionally, in this analysis, the number of anti-VEGF injections was stratified by tertiles, resulting in slightly different group boundaries for BRVO and CRVO/HRVO eyes; it is worth noting that identical boundaries may have been more useful for clinicians to interpret. Finally, study results may not be generalisable to patients outside of England due to differences in global healthcare practices, or patients seeking care from private ophthalmology clinics, where treatment patterns may differ.

This study addresses a knowledge gap in real-world VA outcomes and treatment patterns in RVO, with a large sample size including BRVO and CRVO. A notable strength is the long-term follow-up period to 5 years, in a field where long-term literature is scarce, particularly from multi-site studies. Further, this study explores the impact of socioeconomic status on access to treatment, with a strong representation of patients across IMD deciles. In addition, the Medisoft EMR captures structured data on important vision-related information including VA outcomes and treatment patterns, which improves data quality and completeness. Finally, the study provides important clinical information about the long-term treatment and outcomes of RVO eyes in routine clinical practice, which is valuable in informing the long-term treatment and management of these patients in real-world settings.

## Conclusions

RVO often requires frequent monitoring and long-term management. In this study, VA improvements occurred early but were lower through 60 months of follow-up, although remaining positive. This, coupled with the lower number of injections through the follow-up period, suggests the need for continued monitoring of patients with RVO over time and treatments with better durability to sustain vision improvements with fewer injections. Anti-VEGF therapy was well-tolerated to 60 months, with low incidences of pre-defined safety and severe outcomes throughout the follow-up period. These results add to the growing body of evidence that current single-target IVT anti-VEGF treatments may not be sufficient for long-term management of RVO, with acceptable treatment burden, in a real-world environment.

## Summary

### What was known before

- Macular oedema secondary to retinal vein occlusion (RVO) represents a significant cause of retinovascular disease and can lead to vision loss if not adequately treated.
- Pharmacological treatments for RVO include intravitreally administered anti-vascular endothelial growth factor (anti-VEGF) therapies and steroids.
- Real-world visual acuity outcomes in patients with RVO are often poorer than those observed in clinical trials due to the burden frequent intravitreal injections places on healthcare systems, patients, and caregivers.

### What this study adds

- This real-world, retrospective, multi-site, observational analysis followed eyes with macular oedema secondary to RVO receiving anti-VEFG treatment in England for up to 60 months.
- Visual acuity improvements were observed early but were not maintained over time, with less pronounced improvements in vision observed through 60 months of follow-up compared with the first months following treatment initiation.
- These data suggest that current single-target anti-VEGF therapies may be insufficient for long-term management of RVO, highlighting the need for durable treatment options for patients with RVO providing sustained effectiveness and an acceptable treatment burden.

## Supplementary information

Supplementary Materials

## Data Availability

For up to date details on Roche’s Global Policy on the Sharing of Clinical Information and how to request access to related clinical study documents see here: https://go.roche.com/data_sharing. Anonymised records for individual patients across more than one data source external to Roche cannot, and should not, be linked due to a potential increase in risk of patient re-identification.
