# Supplementary material for: Treatment patterns and long-term outcomes in anti-VEGF-treated macular oedema secondary to retinal vein occlusion: a retrospective observational study
Source: Eye (Lond). 2025 Nov 14;40(1):107–16. doi: 10.1038/s41433-025-04089-2 (PMC12764885; doi:10.1038/s41433-025-04089-2)
Supplement: Supplementary file 1 — Supplementary Materials [file 41433_2025_4089_MOESM1_ESM.docx]

**Treatment patterns and long-term outcomes in anti-VEGF-treated macular oedema secondary to retinal vein occlusion: A retrospective observational study**

**Supplementary Materials**

**Supplementary Methods**

*Medisoft Proprietary Diagnosis Library*

Each term the Medisoft proprietary diagnosis library is linked to an ICD-10 code where applicable.

Eyes were identified if they either had a diagnosis of “branch retinal vein occlusion with macular oedema”, “central retinal vein occlusion with macular oedema”, or “hemi-retinal vein occlusion with macular oedema” (all linked to ICD-10 code H34.8), or they had a diagnosis of “macular oedema” (linked to ICD-10 code H35.8) on or after a diagnosis of a term denoting “branch retinal vein occlusion”, “central retinal vein occlusion”, or “hemi-retinal vein occlusion” (all linked to ICD-10 code H34.8).

*VA Calculations*

VA recorded in Snellen or logMAR was converted to and reported as an approximate Early Treatment Diabetic Retinopathy Study (ETDRS) letter score, rounded to the nearest whole number, using the separate formulae for Snellen and logMAR^1^:

- - ETDRS = 85 + 50 * log_10_(Snellen fraction)
  - ETDRS = 85 - 50 * logMAR

The semi-quantitative values of Count Fingers (CF), Hand Motion (HM), Perception of Light (PL) and No Perception of Light (NPL) were converted to 0 approximate ETDRS letter score, as were any logMAR values worse than 1.68. The correction and notation used when the measurement was recorded were both retained in the database of the Medisoft EHRs and extracted. A correction priority order was implemented to reduce potential bias if a different correction was used at baseline and follow-up intervals for any given patient. At each timepoint, the highest value between habitual correction VA and unaided VA was used, and where no results are available for either of these measures, pinhole VA was used.^2^

*Stratification of Baseline VA*

Baseline VA was stratified using approximate ETDRS letter thresholds. The stratification groups were ≤54 letters and ≥55 letters for BRVO eyes and ≤34 letters, 35−54 letters and ≥55 letters for CRVO/HRVO eyes. The VA threshold used to stratify BRVO eyes was determined based on the mean baseline VA for BRVO eyes (55.3 approximate letters). For CRVO/HRVO eyes, the upper threshold of ≥55 letters was used to retain consistency with the BRVO cohort, while a lower threshold was also required due to the lower baseline VA of these eyes (39.5 approximate letters); the threshold of 35 letters was used for consistency with similar publications.^3–5^ Adjustment was not made for VA as a continuous measure as there is not a linear relationship with baseline VA scores; there is a ceiling effect, as eyes with lower baseline VA are likely to have greater VA gains than those with higher baseline VA.

*Stratification of Number of Anti-VEGF Injections*

Number of anti-VEGF injections through 60 months was stratified by tertiles as follows: for BRVO, low: ≤10 injections, medium: 11–23 injections, and high: ≥24 injections; for CRVO/HRVO, low: ≤11 injections, medium: 12–24 injections, and high: ≥25 injections. These group boundaries were chosen by calculating the tertiles of the number of injections at 60 months for BRVO and CRVO/HRVO eyes, to create groups approximately equal in size, which resulted in slightly different boundaries for BRVO and CRVO/HRVO.

*Treatment Patterns*

In the evaluation of treatment patterns, non-persistence or loss to follow-up was defined as a patient having no recorded visit for a period of ≥12 months (360 days).

*Safety Outcomes*
Safety outcomes were classified as chronic or acute, depending on the outcome type. For chronic outcomes, if a patient had ≥1 event corresponding to the same outcome within a timepoint, only one event was recorded for that timepoint. For acute outcomes, each outcome with a gap of at least 30 days since the previous acute outcome of the same type was tracked. The safety outcomes were classified as chronic or acute as follows:

- Chronic: traumatic cataract, cataract surgery, ocular hypertension, glaucoma, possible progression of retinal ischaemia.
- Acute: retinal neovascularisation, vitreous haemorrhage, other IOI, endophthalmitis, retinal artery occlusion, retinal tear, retinal detachment.

**References**

1. Gregori NZ, Feuer W, Rosenfeld PJ. Novel method for analyzing snellen visual acuity measurements. Retina 2010;30:1046-50.
2. Jaycock, P; Johnston, RL; Taylor, H et al. The Cataract National Dataset electronic multi-centre audit of 55,567 operations: updating benchmark standards of care in the United Kingdom and internationally. Eye (Lond). 2009;23(1):38–49.
3. Hogg, J; Di Simplicio, S; Talks, JS. The temporal relationship of anti-VEGF injections and the development of neovascularisation in central retinal vein occlusion. Invest. Ophthalmol. Vis. Sci. 2019;60(9):2594.
4. Sivaprasad, S; Amoaku, W; Hykin, P et al. The Royal College of Ophthalmologists Guidelines on retinal vein occlusions: executive summary. Eye. 2015;29:1633–1638.
5. Pandya, B; Mihalache, A; Hatamnejad, A et al. The Association between Retinal Thickness Fluctuations and Visual Outcomes under Anti-Vascular Endothelial Growth Factor Therapy: A Systematic Review and Meta-Analysis. Ophthalmologica. 2024;247(4):261–274.

**Supplementary Results**

**Supplementary Table 1.** Distribution of visual acuity notations used at baseline

|  | **Overall** | | **Eyes with VA ≤35 letters at index treatment** | |
| --- | --- | --- | --- | --- |
|  | **BRVO** | **CRVO/HRVO** | **BRVO** | **CRVO/HRVO** |
| Snellen, n (%) | 1,898 (54.1) | 1,886 (52.9) | 290 (51.4) | 708 (47.4) |
| logMAR, n (%) | 898 (25.6) | 877 (24.6) | 163 (28.9) | 387 (25.9) |
| ETDRS letter score, n (%) | 715 (20.4) | 805 (22.6) | 111 (19.7) | 399 (26.7) |
| **Total** | **3,511** | **3,568** | **564** | **1,494** |

|  | **BRVO** | | | | | | | | **CRVO/HRVO** | | | | | | | | | |
| --- | --- | --- | --- | --- | --- | --- | --- | --- | --- | --- | --- | --- | --- | --- | --- | --- | --- | --- |
| **Follow-up time point** | **3 months** | **6 months** | **12 months** | **18 months** | **24 months** | **36 months** | **48 months** | **60 months** | **3 months** | | **6 months** | **12 months** | **18 months** | **24 months** | **36 months** | **48 months** | **60 months** |  |
| Eligible eyes, n | 3,511 | 3,275 | 2,856 | 2,425 | 2,007 | 1,369 | 1,012 | 673 | 3,568 | 3,326 | | 2,879 | 2,425 | 2,055 | 1,420 | 993 | 679 |  |
| **Anti-VEGF agent, n (%)** | | | | | | | | | | | | | | | | | | |
| Aflibercept^a^ only | 1,660 (47.3) | 1,492 (45.6) | 1,238 (43.4) | 994 (41.0) | 756 (37.7) | 428 (31.3) | 260 (25.7) | 134 (19.9) | 2,217 (62.1) | 2,032 (61.1) | | 1,702 (59.1) | 1,378 (56.8) | 1,133 (55.1) | 707 (49.8) | 467 (47.0) | 285 (42.0) |  |
| Ranibizumab^a^ only | 1,128 (33.1) | 1,087 (33.8) | 969 (33.9) | 837 (34.5) | 698 (34.8) | 500 (36.5) | 385 (38.0) | 264 (39.2) | 760 (21.9) | 722 (21.7) | | 647 (22.5) | 557 (23.0) | 470 (22.9) | 331 (23.3) | 229 (23.1) | 166 (24.5) |  |
| Mixed or other anti-VEGF treatment | 698  (19.9) | 675 (20.6) | 634 (22.2) | 580 (23.9) | 543 (27.1) | 434 (31.7) | 360 (35.6) | 269 (40.0) | 530 (15.9) | 522 (15.7) | | 501 (17.4) | 458 (18.9) | 428 (20.8) | 367 (25.9) | 288 (29.0) | 221 (32.6) |  |

**Supplementary Table 2.** Types of anti-VEGF agents used from Month 3 to Month 60 and their prevalence

^a^Eylea^®^ 2 mg was assessed for aflibercept. ^b^Lucentis^®^ was assessed for ranibizumab. Ongavia^®^, a biosimilar of Lucentis^®^, was included in the ‘mixed or other anti-VEGF treatment’ group. BRVO: branch retinal vein occlusion; CRVO: central retinal vein occlusion; HRVO: hemiretinal vein occlusion; N/A: not applicable; VEGF: vascular endothelial growth factor.

**Supplementary Table 3a. Patient demographics and eye characteristics among BRVO eyes with or without VA data at Month 60**

| **Patient demographics** | | **BRVO patients with VA at Month 60** | | **BRVO patients without VA at Month 60** |
| --- | --- | --- | --- | --- |
| Number of **patients** with macular oedema secondary to BRVO between 2013–2023^a^ | | 497 | | 2,972 |
| **Age at index treatment (years)^b^** | | | | |
| Mean (SD) | | 69.4 (10.1) | | 71.8 (12.5) |
| **Sex, n (%)** | | | | |
| Female | | 279 (56.1) | | 1,575 (53.0) |
| Male | | 218 (43.9) | | 1,397 (47.0) |
| **Race/ethnicity, n (%)** | | | | |
| White British | | 437 (87.9) | | 2,328 (78.3) |
| White Irish | | 2 (0.4) | | 17 (0.6) |
| Asian or Asian British | | 9 (1.8) | | 76 (2.6) |
| Black or Black British | | 2 (0.4) | | 18 (0.6) |
| Chinese | | 0 | | 4 (0.1) |
| Any other White background | | 11 (2.2) | | 55 (1.9) |
| Any other mixed background | | 1 (0.2) | | 6 (0.2) |
| Any other ethnic group | | 3 (0.6) | | 17 (0.6) |
| Not stated | | 32 (6.4) | | 451 (15.2) |
| **IMD decile, n (%)** | | | | |
| 1 (most deprived) | | 33 (6.6) | | 233 (7.8) |
| 2 | | 33 (6.6) | | 201 (6.8) |
| 3 | | 33 (6.6) | | 219 (7.4) |
| 4 | | 45 (9.1) | | 286 (9.6) |
| 5 | | 42 (8.5) | | 298 (10.0) |
| 6 | | 51 (10.3) | | 340 (11.4) |
| 7 | | 69 (13.9) | | 331 (11.1) |
| 8 | | 57 (11.5) | | 339 (11.4) |
| 9 | | 50 (10.1) | | 312 (10.5) |
| 10 (least deprived) | | 82 (16.5) | | 397 (13.4) |
| Not stated | | 2 (0.4) | | 16 (0.5) |
| **Eye characteristics** | | **BRVO eyes with VA at Month 60** | | **BRVO eyes without VA at Month 60** |
| Number of **eyes** with macular oedema secondary to BRVO between 2013–2023 | | 497 | | 3,014 |
| **Laterality, n (%)** | | | | |
| Left | 264 (53.1) | | 1,486 (49.3) | |
| Right | 233 (46.9) | | 1,528 (50.7) | |
| **Lens status, n (%)** | | | | |
| Aphakic | 1 (0.2) | | 9 (0.3) | |
| Phakic | 412 (82.9) | | 2,207 (73.2) | |
| Pseudophakic | 56 (11.3) | | 650 (21.6) | |
| Not known | 28 (5.6) | | 148 (4.9) | |
| **Year of diagnosis, n (%)** | | | | |
| 2013 | 21 (4.2) | | 43 (1.4) | |
| 2014 | 70 (14.1) | | 128 (4.3) | |
| 2015 | 82 (16.5) | | 208 (6.9) | |
| 2016 | 126 (25.4) | | 204 (6.8) | |
| 2017 | 134 (27.0) | | 194 (6.4) | |
| 2018 | 64 (12.9) | | 325 (10.8) | |
| 2019 | 0 | | 396 (13.1) | |
| 2020 | 0 | | 377 (12.5) | |
| 2021 | 0 | | 506 (16.8) | |
| 2022 | 0 | | 461 (15.3) | |
| 2023 | 0 | | 172 (5.7) | |
| **Duration since diagnosis at index treatment (days)^b^** | | | | |
| Median (Q1–Q3) | 0 (0–20) | | 0 (0–14) | |
| **Duration of follow-up after index treatment, n (%)^b^** | | | | |
| ≥3 months (≥90 days) | 497 (100) | | 3,014 (100) | |
| ≥6 months (≥180 days) | 497 (100) | | 2,778 (92.2) | |
| ≥12 months (≥360 days) | 497 (100) | | 2,359 (78.3) | |
| ≥18 months (≥540 days) | 497 (100) | | 1,928 (64.0) | |
| ≥24 months (≥720 days) | 497 (100) | | 1,510 (50.1) | |
| ≥36 months (≥1080 days) | 497 (100) | | 872 (28.9) | |
| ≥48 months (≥1440 days) | 497 (100) | | 515 (17.1) | |
| ≥60 months (≥1800 days) | 497 (100) | | 176 (5.8)^c^ | |
| **VA at index treatment (approximate ETDRS letter score)^b^** | | | | |
| ≤35 letters | 53 (10.7) | | 511 (17.0) | |
| ≥70 letters | 138 (27.8) | | 770 (25.6) | |
| ≥85 letters | 11 (2.2) | | 50 (1.7) | |
| **VA at index treatment (approximate ETDRS letter score)^b^** | | | | |
| Median (Q1–Q3) | 61 (50–70) | | 60 (46–70) | |

Demographic data are reported at the individual patient level; eye characteristics are reported at the individual eye level. ^a^Four patients with bilateral BRVO who had Month 60 VA data for one eye but not the other are included in both columns. ^b^Index treatment defined as the first anti-VEGF treatment an eye received following initial diagnosis. ^c^176 eyes were followed up for ≥60 months but did not provide VA data at Month 60. BRVO: branch retinal vein occlusion; ETDRS: Early Treatment Diabetic Retinopathy Study; IMD: Index of Multiple Deprivation; Q1: lower quartile; Q3: upper quartile; SD: standard deviation; VA: visual acuity.

**Supplementary Table 3b. Patient demographics and eye characteristics among CRVO/HRVO eyes with or without VA data at Month 60**

| **Patient demographics** | | **CRVO/HRVO patients with VA at Month 60** | | **CRVO/HRVO patients without VA at Month 60** |
| --- | --- | --- | --- | --- |
| Number of **patients** with macular oedema secondary to CRVO/HRVO between 2013–2023^a^ | | 498 | | 3,022 |
| **Age at index treatment (years)^b^** | | | | |
| Mean (SD) | | 71.5 (10.4) | | 74.0 (12.6) |
| **Sex, n (%)** | | | | |
| Female | | 221 (44.4) | | 1,453 (48.1) |
| Male | | 277 (55.6) | | 1,569 (51.9) |
| **Race/ethnicity, n (%)** | | | | |
| White British | | 427 (85.7) | | 2,502 (82.8) |
| White Irish | | 1 (0.2) | | 14 (0.5) |
| Asian or Asian British | | 9 (1.8) | | 65 (2.2) |
| Black or Black British | | 3 (0.6) | | 24 (0.8) |
| Chinese | | 0 | | 1 (0.0) |
| Any other White background | | 9 (1.8) | | 32 (1.1) |
| Any other mixed background | | 1 (0.2) | | 7 (0.2) |
| Any other ethnic group | | 0 | | 18 (0.6) |
| Not stated | | 48 (9.6) | | 359 (11.9) |
| **IMD decile, n (%)** | | | | |
| 1 (most deprived) | | 40 (8.0) | | 268 (8.9) |
| 2 | | 34 (6.8) | | 214 (7.1) |
| 3 | | 29 (5.8) | | 264 (8.7) |
| 4 | | 42 (8.4) | | 295 (9.8) |
| 5 | | 47 (9.4) | | 287 (9.5) |
| 6 | | 57 (11.5) | | 326 (10.8) |
| 7 | | 51 (10.2) | | 335 (11.1) |
| 8 | | 52 (10.4) | | 332 (11.0) |
| 9 | | 61 (12.3) | | 300 (9.9) |
| 10 (least deprived) | | 82 (16.5) | | 380 (12.6) |
| Not stated | | 3 (0.6) | | 21 (0.7) |
| **Eye characteristics** | | **CRVO/HRVO eyes with VA at Month 60** | | **CRVO/HRVO eyes without VA at Month 60** |
| Number of **eyes** with macular oedema secondary to CRVO/HRVO between 2013–2023 | | 498 | | 3,070 |
| **Laterality, n (%)** | | | | |
| Left | 232 (46.6) | | 1,484 (48.3) | |
| Right | 266 (53.4) | | 1,586 (51.7) | |
| **Lens status, n (%)** | | | | |
| Aphakic | 2 (0.4) | | 6 (0.2) | |
| Phakic | 419 (84.1) | | 2,333 (76.0) | |
| Pseudophakic | 68 (13.7) | | 677 (22.1) | |
| Not known | 9 (1.8) | | 54 (1.8) | |
| **Year of diagnosis, n (%)** | | | | |
| 2013 | 24 (4.8) | | 60 (2.0) | |
| 2014 | 62 (12.5) | | 126 (4.1) | |
| 2015 | 84 (16.9) | | 202 (6.6) | |
| 2016 | 117 (23.5) | | 252 (8.2) | |
| 2017 | 135 (27.1) | | 236 (7.7) | |
| 2018 | 75 (15.1) | | 334 (10.9) | |
| 2019 | 1 (0.2) | | 383 (12.5) | |
| 2020 | 0 | | 404 (13.2) | |
| 2021 | 0 | | 444 (14.5) | |
| 2022 | 0 | | 476 (15.5) | |
| 2023 | 0 | | 153 (5.0) | |
| **Duration since diagnosis at index treatment (days)^b^** | | | | |
| Median (Q1–Q3) | 1 (0–16.75) | | 0 (0–14) | |
| **Duration of follow-up after index treatment, n (%)^b^** | | | | |
| ≥3 months (≥90 days) | 498 (100) | | 3,070 (100) | |
| ≥6 months (≥180 days) | 498 (100) | | 2,828 (92.1) | |
| ≥12 months (≥360 days) | 498 (100) | | 2,381 (77.6) | |
| ≥18 months (≥540 days) | 498 (100) | | 1,927 (62.8) | |
| ≥24 months (≥720 days) | 498 (100) | | 1,557 (50.7) | |
| ≥36 months (≥1080 days) | 498 (100) | | 922 (30.0) | |
| ≥48 months (≥1440 days) | 498 (100) | | 495 (16.1) | |
| ≥60 months (≥1800 days) | 498 (100) | | 181 (5.9)^c^ | |
| **VA at index treatment (approximate ETDRS letter score)^b^** | | | | |
| ≤35 letters | 162 (32.5) | | 1,332 (43.4) | |
| ≥70 letters | 71 (14.3) | | 341 (11.1) | |
| ≥85 letters | 3 (0.6) | | 19 (0.6) | |
| **VA at index treatment (approximate ETDRS letter score)^b^** | | | | |
| Median (Q1–Q3) | 49 (26.5–61) | | 44 (20–60) | |

Demographic data are reported at the individual patient level; eye characteristics are reported at the individual eye level. ^a^Six patients with bilateral CRVO/HRVO who had Month 60 VA data for one eye but not the other are included in both columns. ^b^Index treatment defined as the first anti-VEGF treatment an eye received following initial diagnosis. ^c^181 eyes were followed up for ≥60 months but did not provide VA data at Month 60. CRVO: central retinal vein occlusion; ETDRS: Early Treatment Diabetic Retinopathy Study; HRVO: hemiretinal vein occlusion; IMD: Index of Multiple Deprivation; Q1: lower quartile; Q3: upper quartile; SD: standard deviation; VA: visual acuity.

**Supplementary Table 4a.** Severe outcomes for eyes with BRVO

|  | **Type** | **BRVO eyes** | | | | | | | |
| --- | --- | --- | --- | --- | --- | --- | --- | --- | --- |
| **Follow-up time point** |  | **3**  **months** | **6**  **months** | **12 months** | **18 months** | **24 months** | **36 months** | **48 months** | **60 months** |
| Any safety outcome for which severe outcomes are studied (n) |  | 41 | 60 | 93 | 124 | 131 | 122 | 115 | 94 |
| Any severe outcome |  | 6 (14.6) | 5 (8.3) | 8 (8.6) | 15 (12.1) | 19 (14.5) | 17 (13.9) | 19 (16.5) | 16 (17.0) |
| Severe vision loss following treatment |  | 5 (12.2) | 4 (6.7) | 5 (5.4) | 10 (8.1) | 12 (9.2) | 8 (6.6) | 7 (6.1) | 6 (6.4) |
| Intervention |  | 1 (2.4) | 1 (1.7) | 4 (4.3) | 7 (5.7) | 12 (9.2) | 14 (11.5) | 16 (13.9) | 14 (14.9) |
| Ocular hypertension | Chronic | 6 | 8 | 16 | 24 | 23 | 20 | 23 | 19 |
| Any severe outcome |  | 0 (0.0) | 0 (0.0) | 0 (0.0) | 0 (0.0) | 0 (0.0) | 0 (0.0) | 0 (0.0) | 0 (0.0) |
| Severe vision loss following treatment |  | 0 (0.0) | 0 (0.0) | 0 (0.0) | 0 (0.0) | 0 (0.0) | 0 (0.0) | 0 (0.0) | 0 (0.0) |
| Intervention |  | 0 (0.0) | 0 (0.0) | 0 (0.0) | 0 (0.0) | 0 (0.0) | 0 (0.0) | 0 (0.0) | 0 (0.0) |
| Glaucoma | Chronic | 24 | 30 | 45 | 54 | 57 | 58 | 57 | 47 |
| Any severe outcome |  | 3 (12.5) | 2 (6.7) | 3 (6.7) | 3 (5.6) | 2 (3.5) | 1 (1.7) | 3 (5.3) | 1 (2.1) |
| Severe vision loss following treatment |  | 2 (8.3) | 1 (3.3) | 1 (2.2) | 1 (1.9) | 0 (0.0) | 0 (0.0) | 0 (0.0) | 0 (0.0) |
| Intervention |  | 1 (4.2) | 1 (3.3) | 2 (4.4) | 2 (3.7) | 2 (3.5) | 1 (1.7) | 3 (5.3) | 1 (2.1) |
| Vitreous haemorrhage | Acute | 0 | 0 | 0 | 3 | 4 | 5 | 5 | 4 |
| Any severe outcome |  | 0 (0.0) | 0 (0.0) | 0 (0.0) | 3 (100.0) | 4 (100.0) | 4 (80.0) | 4 (80.0) | 4 (100.0) |
| Severe vision loss |  | 0 (0.0) | 0 (0.0) | 0 (0.0) | 3 (100.0) | 4 (100.0) | 3 (60.0) | 3 (60.0) | 2 (50.0) |
| Intervention (vitrectomy) |  | 0 (0.0) | 0 (0.0) | 0 (0.0) | 0 (0.0) | 1 (25.0) | 2 (40.0) | 2 (40.0) | 3 (75.0) |
| Traumatic cataract | Chronic | 0 | 0 | 0 | 0 | 0 | 0 | 0 | 0 |
| IOI (overall)^a^ | Acute | 3 | 4 | 4 | 4 | 4 | 2 | 1 | 1 |
| Any severe outcome |  | 1 (33.3) | 1 (25.0) | 1 (25.0) | 1 (25.0) | 1 (25.0) | 0 (0.0) | 0 (0.0) | 0 (0.0) |
| Severe vision loss |  | 1 (33.3) | 1 (25.0) | 1 (25.0) | 1 (25.0) | 1 (25.0) | 0 (0.0) | 0 (0.0) | 0 (0.0) |
| Intervention (vitrectomy) |  | 0 (0.0) | 0 (0.0) | 0 (0.0) | 0 (0.0) | 0 (0.0) | 0 (0.0) | 0 (0.0) | 0 (0.0) |
| Endophthalmitis | Acute | 1 | 1 | 2 | 3 | 4 | 4 | 3 | 3 |
| Any severe outcome |  | 1 (100.0) | 1 (100.0) | 2 (100.0) | 3 (100.0) | 4 (100.0) | 4 (100.0) | 3 (100.0) | 3 (100.0) |
| Severe vision loss |  | 1 (100.0) | 1 (100.0) | 2 (100.0) | 3 (100.0) | 4 (100.0) | 3 (75.0) | 2 (66.7) | 2 (66.7) |
| Intervention (vitrectomy) |  | 0 (0.0) | 0 (0.0) | 1 (50.0) | 1 (33.3) | 2 (50.0) | 3 (75.0) | 2 (66.7) | 2 (66.7) |
| RAO (overall) | Acute | 1 | 4 | 7 | 9 | 9 | 7 | 5 | 3 |
| Severe vision loss |  | 0 (0.0) | 0 (0.0) | 0 (0.0) | 0 (0.0) | 0 (0.0) | 0 (0.0) | 0 (0.0) | 0 (0.0) |
| Retinal tear | Acute | 0 | 1 | 1 | 3 | 4 | 4 | 5 | 5 |
| Any severe outcome |  | 0 (0.0) | 0 (0.0) | 0 (0.0) | 1 (33.3) | 2 (50.0) | 2 (50.0) | 3 (60.0) | 3 (60.0) |
| Severe vision loss |  | 0 (0.0) | 0 (0.0) | 0 (0.0) | 0 (0.0) | 0 (0.0) | 0 (0.0) | 0 (0.0) | 1 (20.0) |
| Intervention (photocoagulation, cryopexy, retinopexy) |  | 0 (0.0) | 0 (0.0) | 0 (0.0) | 1 (33.3) | 2 (50.0) | 2 (50.0) | 3 (60.0) | 3 (60.0) |
| Retinal detachment | Acute | 1 | 2 | 3 | 5 | 7 | 6 | 5 | 5 |
| Any severe outcome |  | 0 (0.0) | 0 (0.0) | 1 (33.3) | 3 (60.0) | 5 (71.4) | 5 (83.3) | 5 (100.0) | 5 (100.0) |
| Severe vision loss |  | 0 (0.0) | 0 (0.0) | 0 (0.0) | 1 (20.0) | 2 (28.6) | 2 (33.3) | 2 (40.0) | 1 (20.0) |
| Intervention (vitrectomy, photocoagulation, retinopexy) |  | 0 (0.0) | 0 (0.0) | 1 (33.3) | 3 (60.0) | 5 (71.4) | 5 (83.3) | 5 (100.0) | 5 (100.0) |
| Retinal neovascularisation | Acute | 1 | 2 | 4 | 6 | 6 | 7 | 5 | 3 |
| Any severe outcome |  | 0 (0.0) | 0 (0.0) | 0 (0.0) | 0 (0.0) | 0 (0.0) | 1 (14.3) | 1 (20.0) | 0 (0.0) |
| Severe vision loss following treatment |  | 0 (0.0) | 0 (0.0) | 0 (0.0) | 0 (0.0) | 0 (0.0) | 0 (0.0) | 0 (0.0) | 0 (0.0) |
| Intervention |  | 0 (0.0) | 0 (0.0) | 0 (0.0) | 0 (0.0) | 0 (0.0) | 1 (14.3) | 1 (20.0) | 0 (0.0) |

Data are reported as the percentage of eyes with eligible data at each time point with a severe outcome (sudden vision loss ≥30 approximate ETDRS letters) or need for surgical intervention, occurring after a recorded safety outcome of interest. The safety outcome counts reported are cumulative over follow-up time and are not mutually exclusive over time points, with loss to follow-up accounting for the fluctuations in safety outcome counts observed through 60 months. ^a^Includes uveitis, vitritis, iridocyclitis, chorioretinitis, anterior chamber flare/inflammation, retinal vasculitis, vitreous cells, iritis. BRVO: branch retinal vein occlusion; ETDRS: Early Treatment Diabetic Retinopathy Study; IOI: intraocular inflammation; RAO: retinal artery occlusion.

**Supplementary Table 4b.** Severe outcomes for eyes with CRVO/HRVO

|  | **Type** | **CRVO/HRVO eyes** | | | | | | | |
| --- | --- | --- | --- | --- | --- | --- | --- | --- | --- |
| **Follow-up time point** |  | **3**  **months** | **6**  **months** | **12 months** | **18 months** | **24 months** | **36 months** | **48 months** | **60 months** |
| Any safety outcome for which severe outcomes are studied (n) |  | 106 | 140 | 183 | 217 | 224 | 223 | 195 | 163 |
| Any severe outcome |  | 15 (14.2) | 22 (15.7) | 25 (13.7) | 31 (14.3) | 32 (14.3) | 35 (15.7) | 28 (14.4) | 28 (17.2) |
| Severe vision loss following treatment |  | 12 (11.3) | 14 (10.0) | 13 (7.1) | 18 (8.3) | 19 (8.5) | 16 (7.2) | 15 (7.7) | 16 (9.8) |
| Intervention |  | 4 (3.8) | 11 (7.9) | 16 (8.7) | 17 (7.8) | 18 (8.0) | 25 (11.2) | 18 (9.2) | 18 (11.0) |
| Ocular hypertension | Chronic | 15 | 22 | 36 | 45 | 46 | 48 | 43 | 30 |
| Any severe outcome |  | 0 (0.0) | 2 (9.1) | 2 (5.6) | 2 (4.4) | 2 (4.4) | 2 (4.2) | 2 (4.7) | 2 (6.7) |
| Severe vision loss following treatment |  | 0 (0.0) | 1 (4.6) | 1 (2.8) | 1 (2.2) | 1 (2.2) | 1 (2.1) | 1 (2.3) | 1 (3.3) |
| Intervention |  | 0 (0.0) | 1 (4.6) | 1 (2.8) | 1 (2.2) | 1 (2.2) | 1 (2.1) | 1 (2.3) | 1 (3.3) |
| Glaucoma | Chronic | 52 | 64 | 91 | 104 | 112 | 110 | 91 | 77 |
| Any severe outcome |  | 6 (11.5) | 7 (10.9) | 8 (8.8) | 8 (7.7) | 9 (8.0) | 9 (8.2) | 4 (4.4) | 5 (6.5) |
| Severe vision loss following treatment |  | 5 (9.6) | 6 (9.4) | 6 (6.6) | 5 (4.8) | 6 (5.4) | 4 (3.6) | 4 (4.4) | 4 (5.2) |
| Intervention |  | 1 (1.9) | 2 (3.1) | 3 (3.3) | 4 (3.9) | 4 (3.6) | 6 (5.5) | 1 (1.1) | 2 (2.6) |
| Vitreous haemorrhage | Acute | 17 | 21 | 21 | 24 | 22 | 23 | 19 | 16 |
| Any severe outcome |  | 1 (5.9) | 3 (14.3) | 4 (19.1) | 5 (20.8) | 4 (18.2) | 6 (26.1) | 6 (31.6) | 6 (37.5) |
| Severe vision loss |  | 1 (5.9) | 1 (4.8) | 0 (0.0) | 3 (12.5) | 2 (9.1) | 2 (8.7) | 2 (10.5) | 2 (12.5) |
| Intervention (vitrectomy) |  | 0 (0.0) | 2 (9.5) | 4 (19.1) | 2 (8.3) | 2 (9.1) | 5 (21.7) | 5 (26.3) | 5 (31.3) |
| Traumatic cataract | Chronic | 0 | 0 | 0 | 0 | 0 | 0 | 0 | 0 |
| IOI (overall)^a^ | Acute | 3 | 5 | 6 | 8 | 7 | 9 | 9 | 9 |
| Any severe outcome |  | 1 (33.3) | 1 (20.0) | 1 (16.7) | 2 (25.0) | 2 (28.6) | 3 (33.3) | 3 (33.3) | 3 (33.3) |
| Severe vision loss |  | 1 (33.3) | 1 (20.0) | 1 (16.7) | 2 (25.0) | 2 (28.6) | 2 (22.2) | 2 (22.2) | 2 (22.2) |
| Intervention (vitrectomy) |  | 0 (0.0) | 0 (0.0) | 0 (0.0) | 0 (0.0) | 0 (0.0) | 1 (11.1) | 1 (11.1) | 1 (11.1) |
| Endophthalmitis | Acute | 3 | 4 | 4 | 5 | 6 | 4 | 4 | 4 |
| Any severe outcome |  | 3 (100.0) | 4 (100.0) | 4 (100.0) | 5 (100.0) | 6 (100.0) | 4 (100.0) | 4 (100.0) | 4 (100.0) |
| Severe vision loss |  | 3 (100.0) | 4 (100.0) | 4 (100.0) | 5 (100.0) | 6 (100.0) | 4 (100.0) | 4 (100.0) | 4 (100.0) |
| Intervention (vitrectomy) |  | 0 (0.0) | 2 (50.0) | 3 (75.0) | 3 (60.0) | 4 (66.7) | 3 (75.0) | 3 (75.0) | 3 (75.0) |
| RAO (overall) | Acute | 5 | 7 | 6 | 7 | 8 | 5 | 6 | 5 |
| Severe vision loss |  | 0 (0.0) | 0 (0.0) | 0 (0.0) | 0 (0.0) | 0 (0.0) | 0 (0.0) | 0 (0.0) | 0 (0.0) |
| Retinal tear | Acute | 1 | 1 | 2 | 3 | 3 | 3 | 2 | 2 |
| Any severe outcome |  | 1 (100.0) | 1 (100.0) | 1 (50.0) | 2 (66.7) | 2 (66.7) | 2 (66.7) | 1 (50.0) | 1 (50.0) |
| Severe vision loss |  | 0 (0.0) | 0 (0.0) | 0 (0.0) | 0 (0.0) | 0 (0.0) | 0 (0.0) | 0 (0.0) | 0 (0.0) |
| Intervention (photocoagulation, cryopexy, retinopexy) |  | 1 (100.0) | 1 (100.0) | 1 (50.0) | 2 (66.7) | 2 (66.7) | 2 (66.7) | 1 (50.0) | 1 (50.0) |
| Retinal detachment | Acute | 0 | 1 | 2 | 2 | 2 | 4 | 2 | 3 |
| Any severe outcome |  | 0 (0.0) | 1 (100.0) | 2 (100.0) | 2 (100.0) | 2 (100.0) | 3 (75.0) | 2 (100.0) | 3 (100.0) |
| Severe vision loss |  | 0 (0.0) | 0 (0.0) | 0 (0.0) | 0 (0.0) | 0 (0.0) | 1 (25.0) | 0 (0.0) | 1 (33.3) |
| Intervention (vitrectomy, photocoagulation, retinopexy) |  | 0 (0.0) | 1 (100.0) | 2 (100.0) | 2 (100.0) | 2 (100.0) | 3 (75.0) | 2 (100.0) | 3 (100.0) |
| Retinal neovascularisation | Acute | 2 | 3 | 3 | 4 | 3 | 3 | 4 | 3 |
| Any severe outcome |  | 2 (100.0) | 2 (66.7) | 2 (66.7) | 3 (75.0) | 3 (100.0) | 3 (100.0) | 3 (75.0) | 1 (33.3) |
| Severe vision loss following treatment |  | 1 (50.0) | 0 (0.0) | 0 (0.0) | 0 (0.0) | 0 (0.0) | 0 (0.0) | 0 (0.0) | 0 (0.0) |
| Intervention |  | 2 (100.0) | 2 (66.7) | 2 (66.7) | 3 (75.0) | 3 (100.0) | 3 (100.0) | 3 (75.0) | 1 (33.3) |

Data are reported as the percentage of eyes with eligible data at each time point with a severe outcome (sudden vision loss ≥30 approximate ETDRS letters) or need for surgical intervention, occurring after a recorded safety outcome of interest. The safety outcome counts reported are cumulative over follow-up time and are not mutually exclusive over time points, with loss to follow-up accounting for the fluctuations in safety outcome counts observed through 60 months. ^a^Includes uveitis, vitritis, iridocyclitis, chorioretinitis, anterior chamber flare/inflammation, retinal vasculitis, vitreous cells, iritis. CRVO: central retinal vein occlusion; ETDRS: Early Treatment Diabetic Retinopathy Study; HRVO: hemiretinal vein occlusion; IOI: intraocular inflammation; RAO: retinal artery occlusion.

**Supplementary Figure 1.** Attrition in the BRVO and CRVO/HRVO eye population


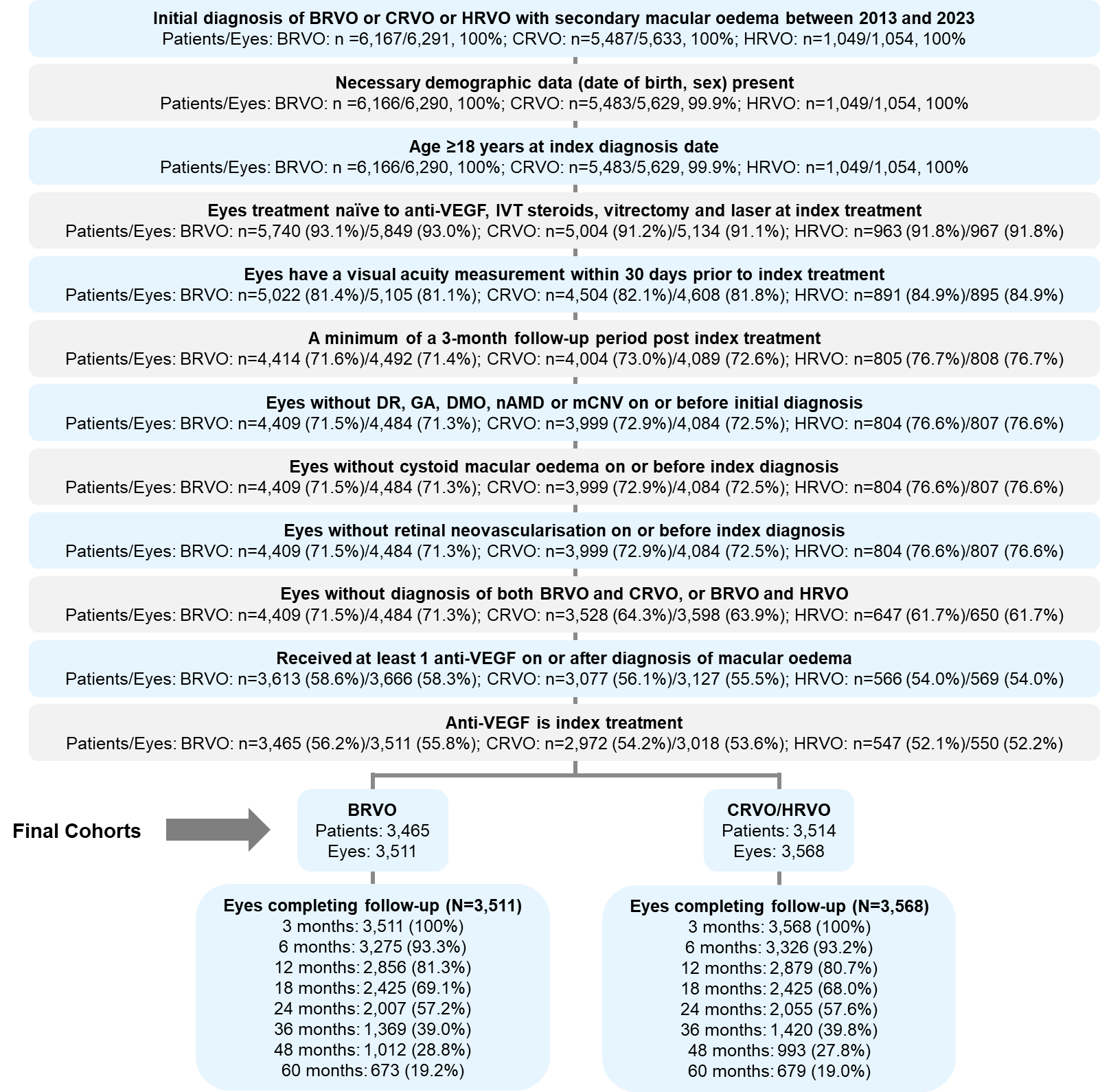


Attrition is described from the total number of patient eyes identified with a diagnosis of BRVO, CRVO or HRVO with secondary macular oedema between 1^st^ January 2013 and the date of data extraction (23^rd^–29^th^ August 2023). BRVO: branch retinal vein occlusion; CRVO: central retinal vein occlusion; DMO: diabetic macular oedema; DR: diabetic retinopathy; GA: geographic atrophy; HRVO: hemiretinal vein occlusion; IVT: intravitreal; mCNV: myopic choroidal neovascularisation; nAMD: neovascular age-related macular degeneration; VEGF: vascular endothelial growth factor.

**Supplementary Figure 2.** VA at index in BRVO and CRVO/HRVO eyes, stratified by IMD deciles


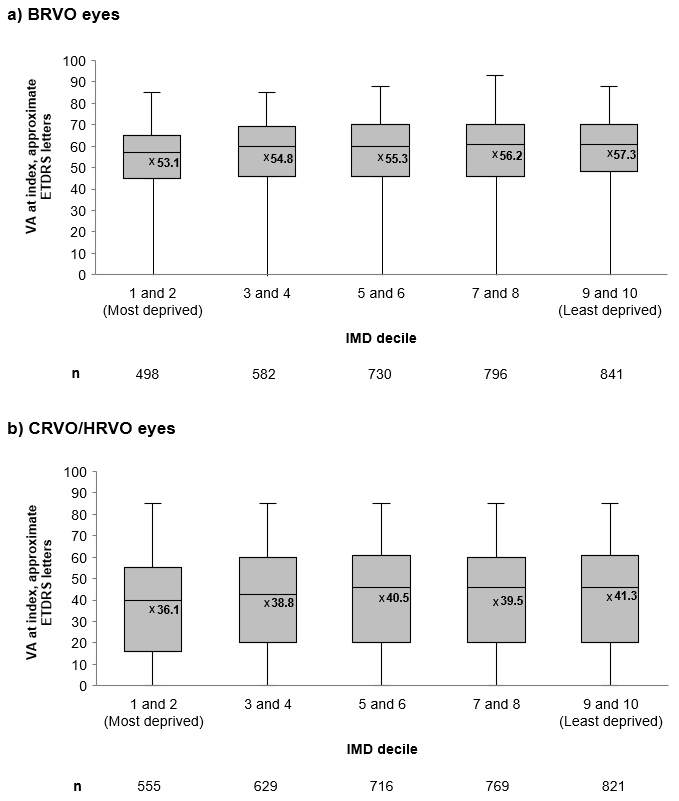


A lower IMD decile represents greater neighbourhood-level deprivation; deciles were analysed in 5 categories of 2 deciles each. x represents the mean value. n numbers represent the number of eyes with a VA measurement at index. BRVO: branch retinal vein occlusion; CRVO: central retinal vein occlusion; ETDRS: Early Treatment Diabetic Retinopathy Study; HRVO: hemiretinal vein occlusion; IMD: Index of Multiple Deprivation; VA: visual acuity.

**Supplementary Figure 3**. Percentage of eyes lost to follow-up at 60 months, stratified by IMD deciles

**
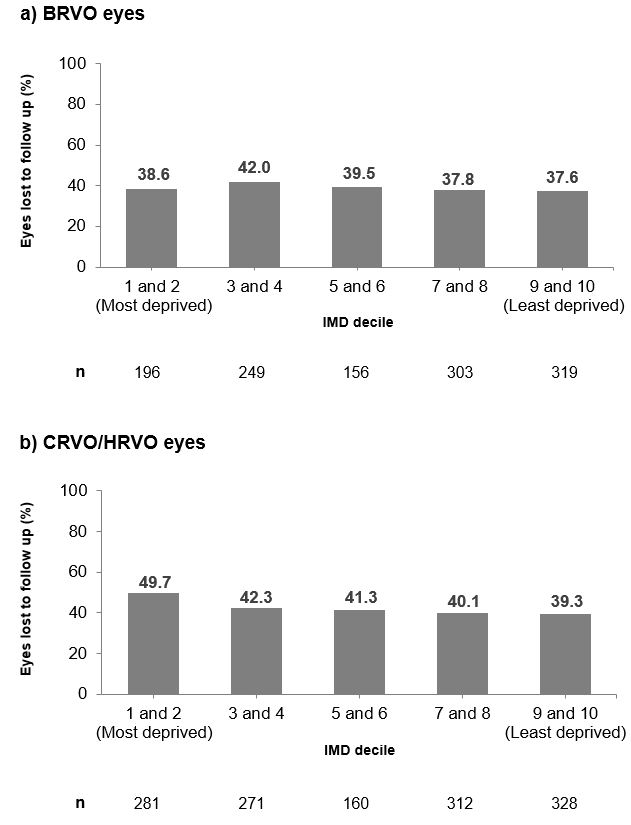
**

A lower IMD decile represents greater neighbourhood-level deprivation; deciles were analysed in 5 categories of 2 deciles each. BRVO: branch retinal vein occlusion; CRVO: central retinal vein occlusion; HRVO: hemiretinal vein occlusion; IMD: Index of Multiple Deprivation.

**Supplementary Figure 4**. VA change from index treatment through 60 months, stratified by loading phase completion


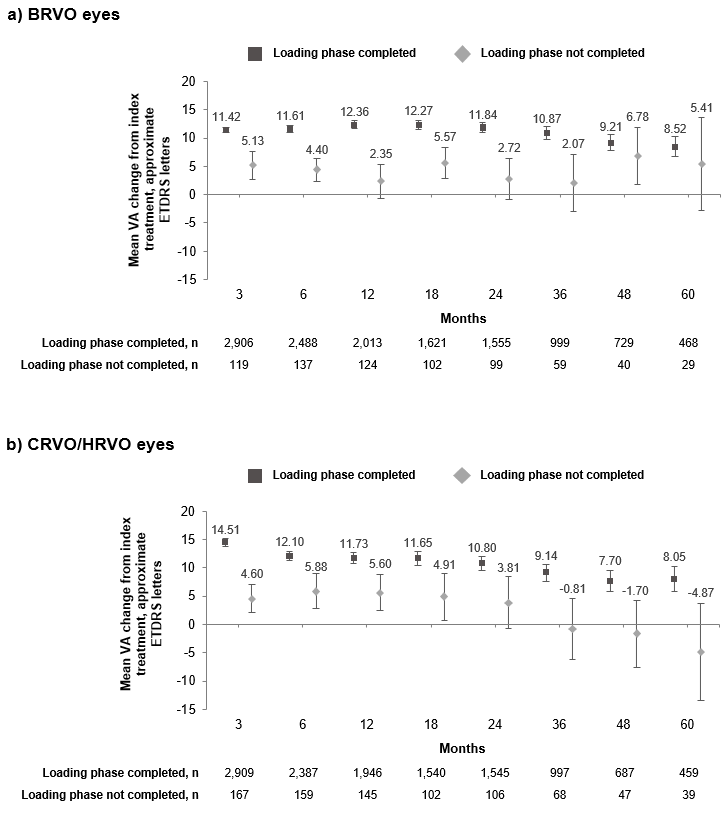


A complete loading dose was defined as ≥3 anti-VEGF injections within 120 days after index; an incomplete loading dose was defined as <3 anti-VEGF infections with 120 days after index. Error bars represent 95% confidence intervals; n numbers represent the number of eyes with a VA measurement at index and each individual time point. BRVO: branch retinal vein occlusion; CRVO: central retinal vein occlusion; ETDRS: Early Treatment Diabetic Retinopathy Study; HRVO: hemiretinal vein occlusion; VA: visual acuity; VEGF: vascular endothelial growth factor.

**Supplementary Figure 5.** Percentage of eyes maintaining VA ≥70 approximate ETDRS letters from baseline through 60 months

**
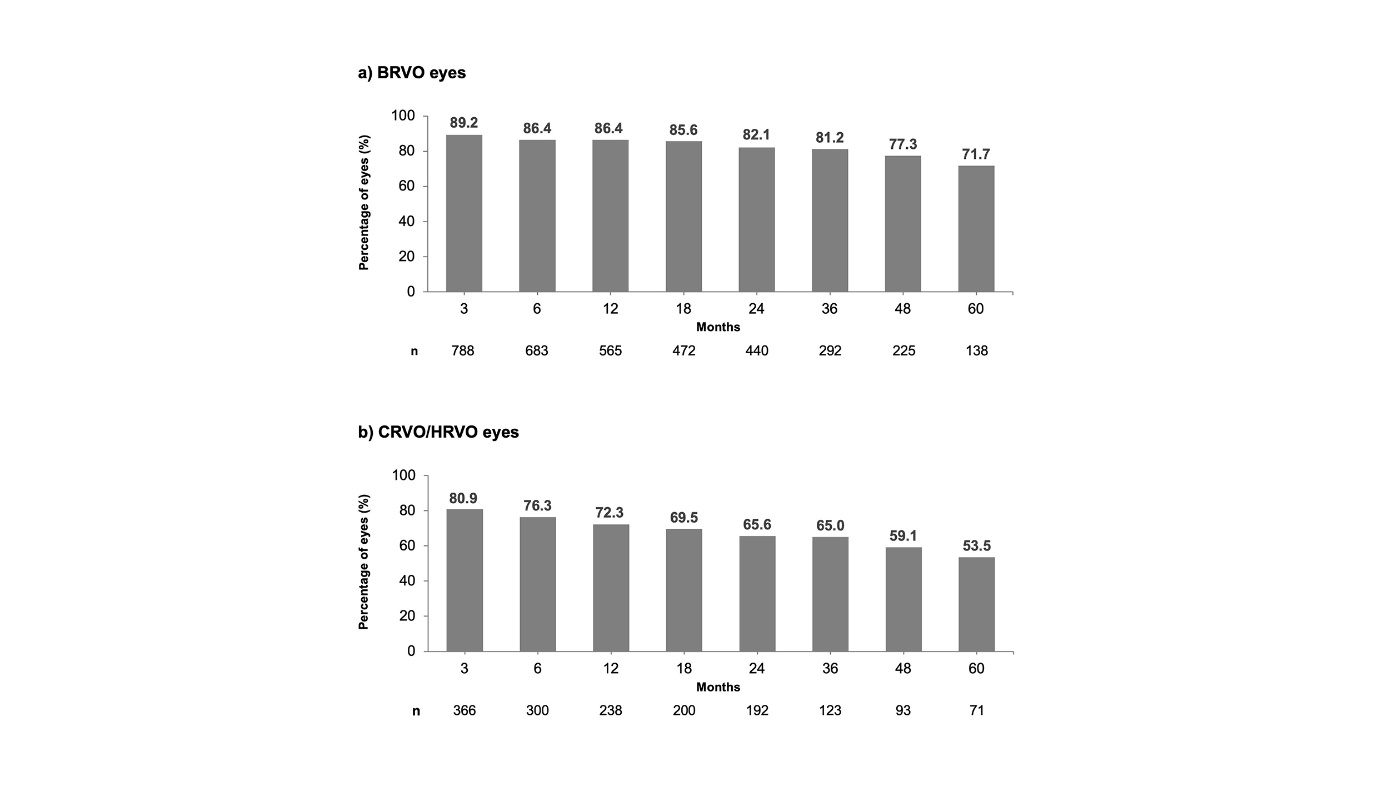
**

n represents the number of eyes with VA ≥70 approximate ETDRS letters at index. BRVO: branch retinal vein occlusion; CRVO: central retinal vein occlusion; ETDRS: Early Treatment Diabetic Retinopathy Study; HRVO: hemiretinal vein occlusion; VA: visual acuity**.**

**Supplementary Figure 6.** Percentage of BRVO and CRVO/HRVO eyes with VA gains of ≥15 approximate ETDRS letters from index treatment through 60 months


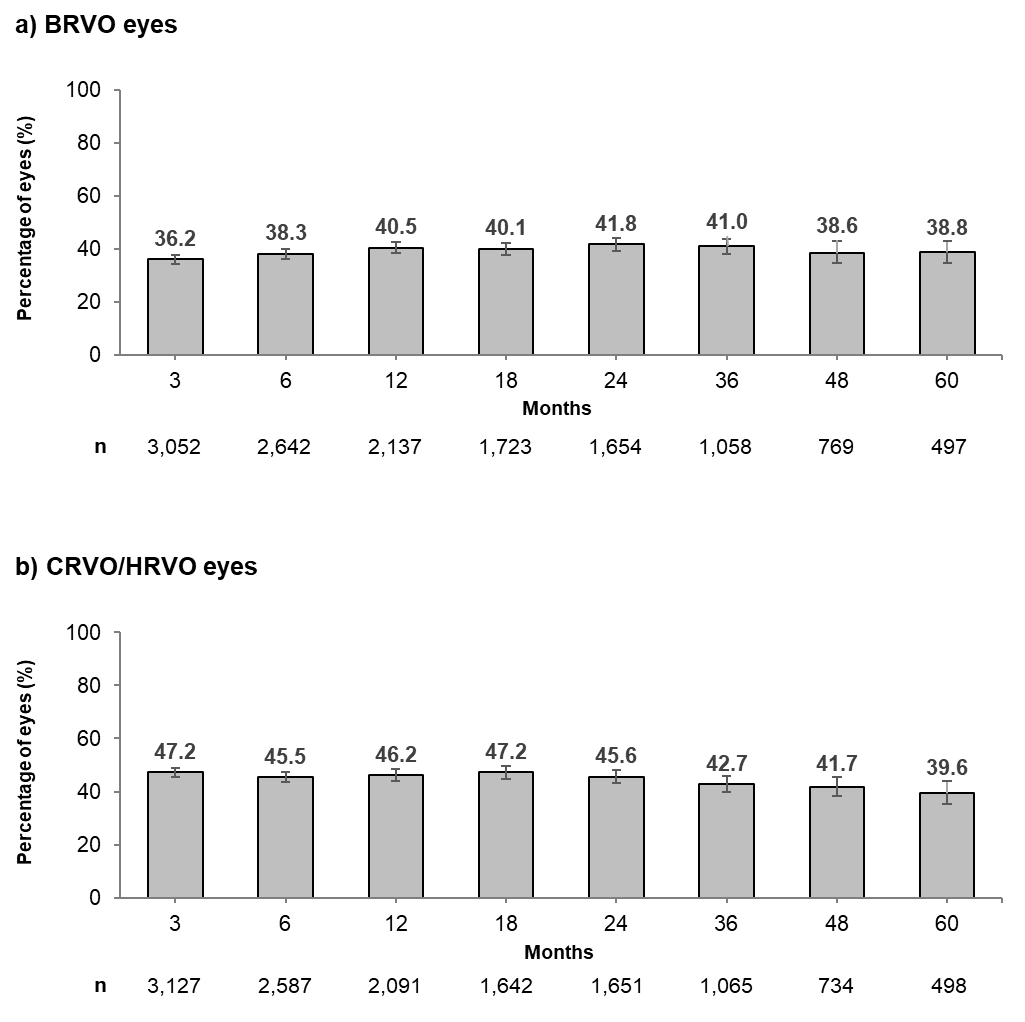


Error bars represent 95% confidence intervals; n represents the number of eyes with a VA measurement at index and at each follow-up timepoint of interest. BRVO: branch retinal vein occlusion; CRVO: central retinal vein occlusion; ETDRS: Early Treatment Diabetic Retinopathy Study; HRVO: hemiretinal vein occlusion; VA: visual acuity.

**Supplementary Figure 7.** Percentage of BRVO and CRVO/HRVO eyes with avoidance of loss of VA ≥15 approximate ETDRS letters from index treatment through 60 months


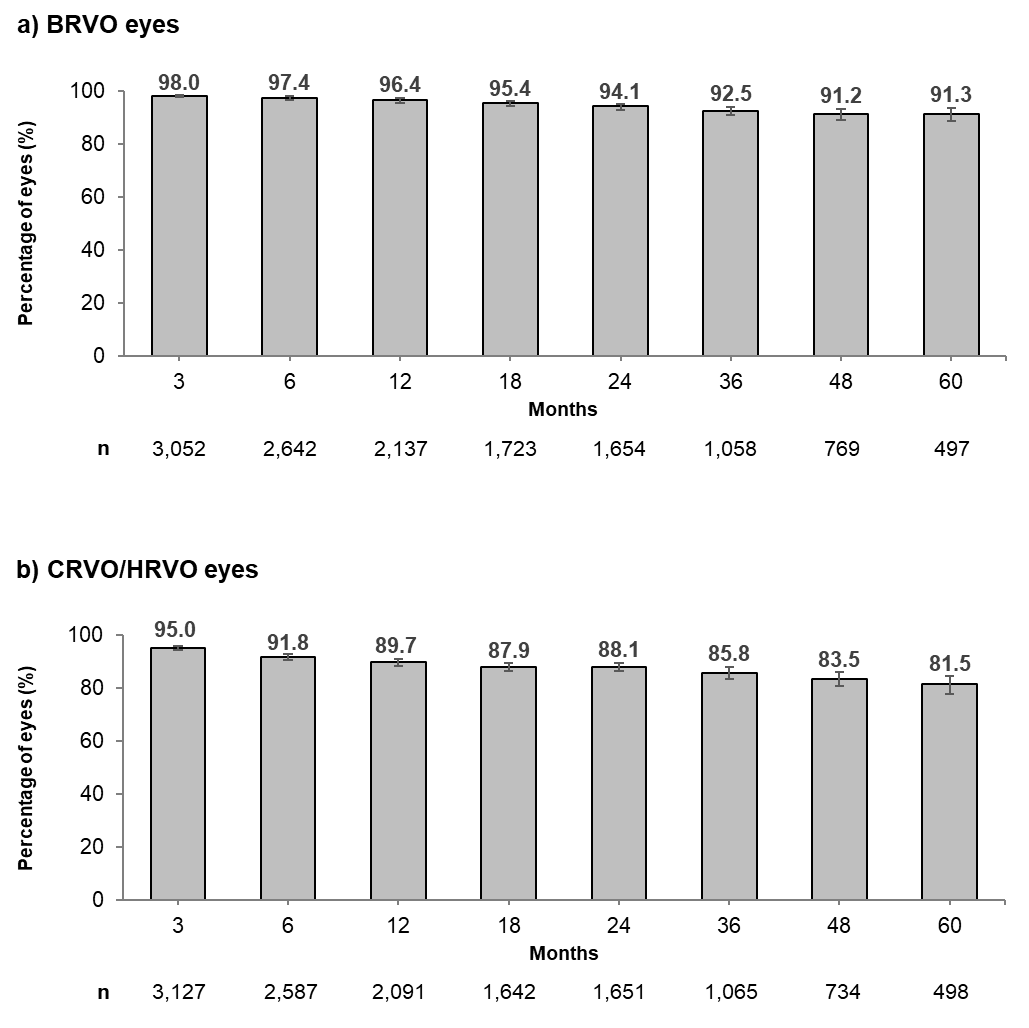


Error bars represent 95% confidence intervals; n represents the number of eyes with a VA measurement at index and at each individual time point. BRVO: branch retinal vein occlusion; CRVO: central retinal vein occlusion; ETDRS: Early Treatment Diabetic Retinopathy Study; HRVO: hemiretinal vein occlusion; VA: visual acuity.

**Supplementary Figure 8.** Percentage of BRVO and CRVO/HRVO eyes that received IVT steroids and macular laser treatment through 60 months

**
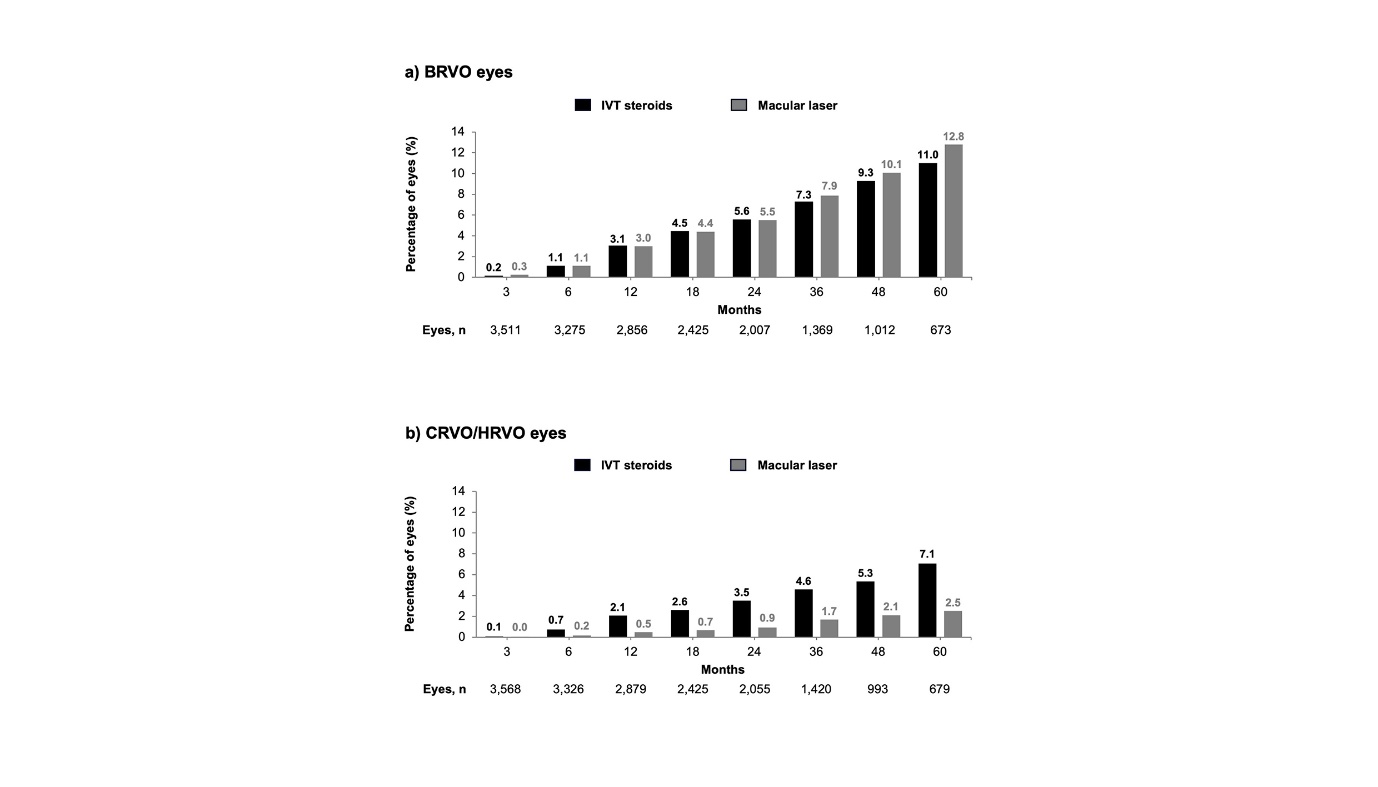
**

BRVO: branch retinal vein occlusion; CRVO: central retinal vein occlusion; HRVO: hemiretinal vein occlusion; IVT: intravitreal.

**Supplementary Figure 9.** Number of anti-VEGF injections received by Month 60, stratified by IMD deciles


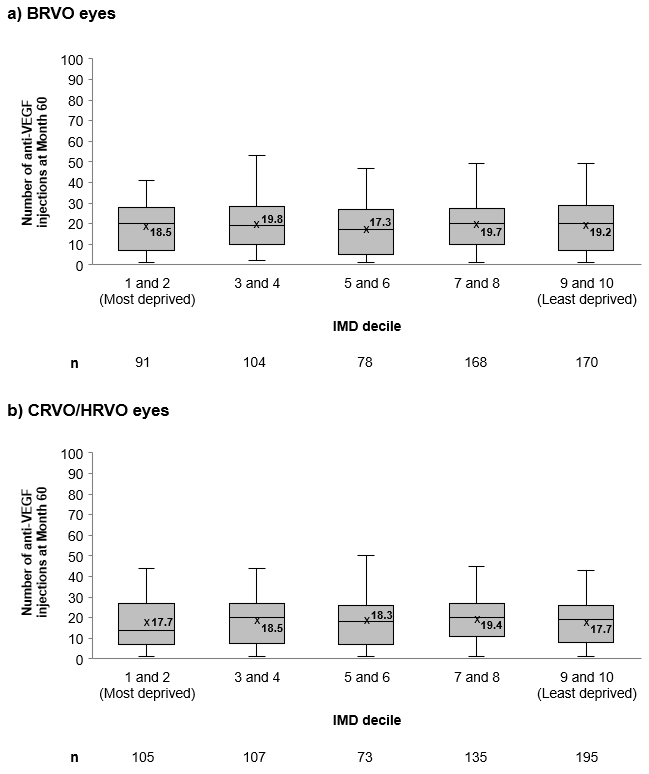


A lower IMD decile represents greater neighbourhood-level deprivation; deciles were analysed in 5 categories of 2 deciles. x represents the mean value. n numbers represent the number of eyes with anti-VEGF treatment. BRVO: branch retinal vein occlusion; CRVO: central retinal vein occlusion; HRVO: hemiretinal vein occlusion; IMD: Index of Multiple Deprivation; VEGF: vascular endothelial growth factor.`
